# Magneto-acoustic protein nanostructures for non-invasive imaging of tissue mechanics in vivo

**DOI:** 10.1038/s41563-023-01688-w

**Published:** 2023-10-16

**Authors:** Whee-Soo Kim, Sungjin Min, Su Kyeom Kim, Sunghwi Kang, Soohwan An, Ernesto Criado-Hidalgo, Hunter Davis, Avinoam Bar-Zion, Dina Malounda, Yu Heun Kim, Jae-Hyun Lee, Soo Han Bae, Jin Gu Lee, Minsuk Kwak, Seung-Woo Cho, Mikhail G. Shapiro, Jinwoo Cheon

**Affiliations:** 1 Center for Nanomedicine, Institute for Basic Science (IBS), Seoul, Republic of Korea; 2 Division of Chemistry and Chemical Engineering, California Institute of Technology, Pasadena, CA USA; 3 Department of Nano Biomedical Engineering (NanoBME), Advanced Science Institute, Yonsei University, Seoul, Republic of Korea; 4 Department of Biotechnology, Yonsei University, Seoul, Republic of Korea; 5 Department of Chemistry, Yonsei University, Seoul, Republic of Korea; 6 Severance Biomedical Science Institute, Yonsei Biomedical Research Institute, Yonsei University College of Medicine, Seoul, Republic of Korea; 7 Severance Biomedical Science Institute, Graduate School of Medical Science, Brain Korea 21 Project, Yonsei University College of Medicine, Seoul, Republic of Korea; 8 Department of Thoracic and Cardiovascular Surgery, Severance Hospital, Yonsei University College of Medicine, Seoul, Republic of Korea; 9 Andrew and Peggy Cherng Department of Medical Engineering, California Institute of Technology, Pasadena, CA USA; 10 Howard Hughes Medical Institute, Pasadena, CA USA

**Keywords:** Biomaterials - proteins, Nanostructures, Ultrasound

## Abstract

Measuring cellular and tissue mechanics inside intact living organisms is essential for interrogating the roles of force in physiological and disease processes. Current agents for studying the mechanobiology of intact, living organisms are limited by poor light penetration and material stability. Magnetomotive ultrasound is an emerging modality for real-time in vivo imaging of tissue mechanics. Nonetheless, it has poor sensitivity and spatiotemporal resolution. Here we describe magneto-gas vesicles (MGVs), protein nanostructures based on gas vesicles and magnetic nanoparticles that produce differential ultrasound signals in response to varying mechanical properties of surrounding tissues. These hybrid nanomaterials significantly improve signal strength and detection sensitivity. Furthermore, MGVs enable non-invasive, long-term and quantitative measurements of mechanical properties within three-dimensional tissues and in vivo fibrosis models. Using MGVs as novel contrast agents, we demonstrate their potential for non-invasive imaging of tissue elasticity, offering insights into mechanobiology and its application to disease diagnosis and treatment.

## Abstract (web summary)

Magneto-gas vesicles, protein nanostructures with enhanced ultrasound signal and sensitivity, enable the non-invasive, long-term and quantitative monitoring of the mechanics of three-dimensional tissues and animals.

## Main

Tissue mechanical properties play a crucial role in various cellular processes, including morphogenesis, tissue homeostasis and disease progression^1^. Among these properties, tissue stiffness is notably altered in numerous pathological conditions such as cancer, diabetes, cardiovascular disease and fibrosis^1^. However, accurately measuring localized tissue stiffness within deep, living three-dimensional (3D) tissues remains challenging^2^. Existing techniques primarily focus on subcellular measurements, are limited to in vitro cultures or dissected tissues and are unable to capture local variations in tissue stiffness^3–8^. Ultrasound-based shear-wave elastography offers non-invasive and quantitative assessment of in vivo tissue stiffness, but its resolution may be limited in detecting small tumours, resolving boundaries between tumours and surrounding tissue and assessing wave speed fluctuation in different tissue types^9^. Recent advances in injectable and deformable materials have enabled the measurement of local mechanical properties in intact soft tissues, but these methods often rely on fluorescent probes and optical imaging, making deployment in deep tissues challenging and limiting long-term measurements due to sensitivity to local factors such as pH and temperature^10–13^. Thus, improved methodologies to accurately measure tissue mechanics in vivo are required.

Magnetomotive ultrasound imaging (MMUS) has emerged as a potential method for measuring tissue mechanics in vivo, but faces challenges due to suboptimal contrast agents resulting in limited resolution and poor sensitivity^14–16^. To address these limitations, we combined gas vesicles (GVs)—air-filled protein nanostructures with highly sensitive ultrasound contrast^17–19^—with superparamagnetic nanoparticles (MNPs) to develop a new class of hybrid protein nanostructures called magneto-GVs (MGVs). Chemical linking of MNPs to GVs significantly improves signal strength and sensitivity compared with conventional MMUS contrast agents. We hypothesize that as the mobility of MNPs is affected by the surrounding mechanical properties, MGVs dynamically alter MMUS contrast in response to tissue mechanics, enabling non-invasive and quantitative measurement of tissue stiffness in vivo. We demonstrate that MGVs have the potential for long-term disease monitoring and drug screening in 3D organoid and in vivo fibrosis models due to their excellent stability, enabling robust and reproducible imaging.

## Synthesis of MGVs with improved imaging contrast

**Fig. 1:**
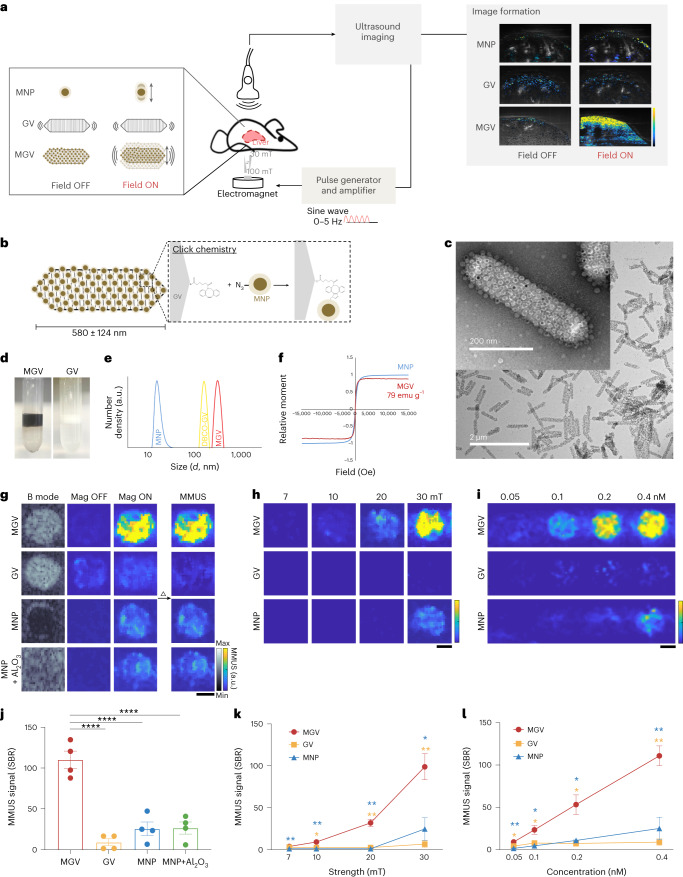
Development and characterization of the MMUS imaging system and MGVs. **a**, Schematic illustration showing the set-up and working principle of the MGV-based MMUS imaging system. **b**, Schematic illustration of conjugating MNPs to GVs using click chemistry to form MGVs. **c**, TEM image of fabricated MGVs. **d**, Images of MGVs and GVs after buoyancy purification. **e**, The hydrodynamic size of MGVs, MNPs and GVs with functionalized DBCO by dynamic light scattering. **f**, The magnetic moment of MGVs and MNPs measured by a vibrating-sample magnetometer. **g**, Comparison of MMUS imaging between MGVs, GVs and MNPs in 0.1% (w/v) agarose phantom using 0.4 nM MGVs, where 0.4 nM MGVs represent 456 pM of GVs and 76 nM of MNPs. *n* = 4 independent experiments. **h**, Magnetic-strength-dependent MMUS images obtained from 7 mT to 30 mT using 0.4 nM MGVs. *n* = 3 independent experiments. **i**, Concentration-dependent MMUS images of different groups. *n* = 3 independent experiments. **j**–**l**, SBR quantification of MMUS images from different cohorts (*P* (left–right) = 0.000006564, 0.000040719, 0.000045239) (**j**), with varying magnetic strength (*P* (blue, left–right) = 0.005394, 0.002267, 0.002239, 0.022173; *P* (yellow, left–right) = 0.590133, 0.012733, 0.003060, 0.004556) (**k**) and concentration (*P* (blue, left–right) = 0.004505, 0.020735, 0.021156, 0.008343; *P* (yellow, left–right) = 0.029392, 0.044301, 0.018321, 0.001178) (**l**) for the same conditions as in **g**–**i**. Scale bars in **g**–**i**, 1 mm. Min and max on the parula (MMUS) and grey (B-mode) colour bars represent 0 and 10,000 arbitrary units, respectively. In **j**–**l** lines and error bars represent mean ± s.e.m., and significance was determined using one-way analysis of variance (ANOVA) with Tukey’s multiple-comparisons test (**j**) and a multiple unpaired two-sided *t*-test (**k**,**l**): *P < 0.05, **P < 0.01, ***P < 0.001, ****P < 0.0001. Source data

To determine whether the combination of particles with high acoustic contrast and strong superparamagnetism can improve MMUS imaging capability, we synthesized magnetic nanoparticle-conjugated gas vesicles (MGVs). We established the MMUS imaging system and optimized the system’s magnetic properties using standard magnetic microparticles (Fig. 1a and Supplementary Fig. 1). Based on the MMUS signal intensity, magnetic parameters of 5 Hz, 30 mT and sine wave were chosen as the frequency, maximum field strength and temporal pattern of the electromagnetic field, respectively (Supplementary Fig. 1a–c). Zinc-doped iron oxide MNPs were synthesized and functionalized with azide groups, as previously reported^20^. We purified GVs from the cyanobacterium *Anabaena flos-aquae*^21^, and added dibenzocyclooctyne (DBCO) to the GV protein surface through an NH_2_–NHS reaction. MGVs were finally synthesized by conjugating azide-functionalized MNPs and DBCO-GVs via click chemistry (Fig. 1b). After a 4 h conjugation reaction, MGVs could be isolated from a suspension of unbound MNPs using buoyancy purification (Fig. 1d). MGVs had a hydrodynamic diameter of approximately 494.1 ± 11.5 nm, while functionalized GVs had a diameter of approximately 272 ± 4.5 nm (Fig. 1e). MGVs were stable in different media conditions or during prolonged storage (Supplementary Fig. 2a,b). The conjugation ratio of MNPs to GVs was approximately 186 MNPs per GV (Fig. 1c and Supplementary Fig. 3). The magnetic moment of MGVs was 79 emu g^−1^, which was comparable to that of MNPs (Fig. 1f).

We next assessed the ability of MGV protein nanostructures to produce robust MMUS signals in response to applied magnetic fields. Using the optimized magnetic conditions identified above, we performed a head-to-head comparison of GVs, MNPs and MGVs with MMUS imaging in agarose phantoms. The concentrations of all materials were matched based on inductively coupled plasma mass spectrometry (ICPMS) measurements and B-mode imaging (Fig. 1g and Supplementary Fig. 3). MGVs showed superior sensitivity and spatiotemporal control compared with other nanomaterials, while MMUS imaging retained the spatial resolution of conventional B-mode ultrasound (Supplementary Fig. 4). Furthermore, despite differences in hydrodynamic size possibly attributed to MNP conjugation, GVs and MGVs showed similar acoustic responses, suggesting the differences in MMUS signals are not due to distinct sound-scattering properties (Supplementary Fig. 5). Magnetic stimulation of MGVs resulted in robust time-locked ultrasound signals, which ceased when the magnetic field was removed (Fig. 1g). The signal-to-background ratio (SBR) of the average pixel intensity was used to quantify MMUS signals. MGVs achieved a 12-fold higher magnetic-field-dependent signal intensity change (Δ, SBR = 110.1 ± 21.3) than GVs (9.1 ± 8.7) (Fig. 1g–j). In addition, MGVs produced a significant increase in signal compared to MNPs (25.6 ± 16.6) or MNPs with tissue-mimicking materials (Al_2_O_3_) (26.5 ± 14.9), suggesting the ability of GVs conjugated to MNPs to transduce magnetic stimulation and produce ultrasound contrast for improved MMUS sensitivity (Fig. 1g–j).

To evaluate the performance limits of MGV-based MMUS imaging, we imaged dispersions containing MGVs, GVs and MNPs at a fixed concentration of 0.4 nM in 0.1% agarose while applying magnetic fields of increasing strength (7–30 mT). MGVs exhibited a robust magnetic MMUS response, with detectable signals from 10 mT to 30 mT. In contrast, GVs showed no visible contrast, while MNPs produced weaker signals only at ≥30 mT (Fig. 1h–k). To assess the sensitivity of the MGV-based approach relative to other nanomaterials, we performed MMUS imaging of a concentration series of MGVs, GVs and MNPs in agarose phantoms with a constant 30 mT magnetic field. MGVs produced significantly higher MMUS signals than the other control materials when the concentration was in the range of 0.05–0.4 nM (Fig. 1i–l). MGVs attained an excellent SBR with a limit of detection of 0.05 nM, representing >8-fold enhancement in sensitivity relative to conventional MNP-based MMUS imaging (Fig. 1i–l). Comparing our results to previous MNP-based MMUS imaging, MGVs showed unique sensitivity to low MNP concentrations and weak magnetic fields (Supplementary Fig. 6). Altogether, these results validate MGVs as novel contrast agents for MMUS imaging with enhanced ultrasound contrast and detection sensitivity.

## Dynamic range of stiffness measurement with MGVs

**Fig. 2:**
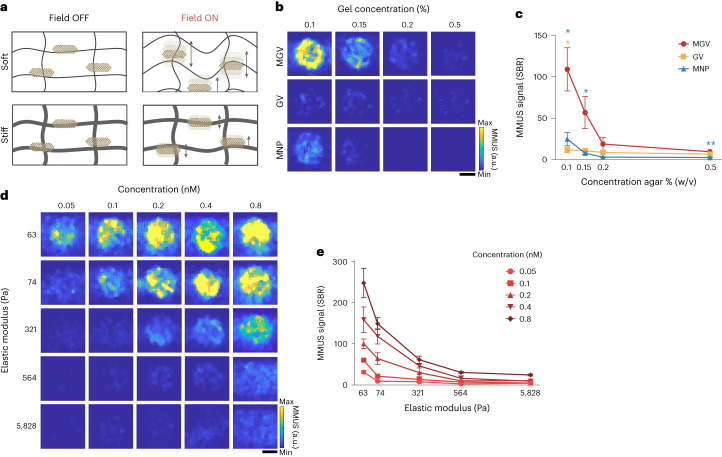
Stiffness-dependent MMUS imaging. **a**, Schematic illustration showing the movement of MGVs inside soft and stiff materials when an applied magnetic field is off and on. **b**,**c**, MMUS images (**b**) and SBR quantification (**c**) of agarose-concentration-dependent movement of MGVs ranging from 0.1% to 0.5% (w/v) agarose. *P* (blue, left–right) = 0.021665, 0.044665, 0.082836, 0.004661; *P* (yellow, left–right) = 0.010210, 0.054968, 0.228487, 0.231079. *n* = 4 independent experiments. **d**,**e**, MMUS images (**d**) and SBR quantification (**e**) based on different values of elastic modulus and concentration. *n* = 5 independent experiments. Scale bars, 1 mm. Min and max on the colour bars represent 0 and 10,000 arbitrary units, respectively. Lines and error bars represent mean ± s.e.m., and significance was determined using a multiple unpaired two-sided *t*-test (**c**) and one-way ANOVA with Tukey’s multiple-comparisons test (**e**): *P < 0.05, **P < 0.01, ***P < 0.001; ****P < 0.0001. Source data

The material stiffness influences the magnetically induced motion of MNPs^15^. Whereas softer materials would allow more MGV movement and induce stronger ultrasound scattering, stiffer materials would restrict MGV motion in response to applied magnetic fields, leading to a decrease in MMUS signal (Fig. 2a). To test the ability of MGVs to quantitatively measure the stiffness of surrounding materials, we performed MMUS imaging in vitro of MGVs embedded in agarose phantoms with varying elastic modulus, ranging from 74 Pa to 5,828 Pa (Fig. 2b). As predicted, we observed an inverse relationship of MGV signals as a function of increasing elastic modulus of the agarose phantoms. At fixed MGV concentrations (OD_500_ = 4, 0.4 nM) and magnetic field (30 mT), MGVs in 0.1% agarose (SBR = 109.1 ± 52.3) produced a 2- and 6-fold greater MMUS signal intensity change than in 0.15% agarose (56.9 ± 38.8) and 0.2% agarose (18.7 ± 15.3), respectively, while generating negligible signal in 0.5% agarose phantom (9.3 ± 3.1) (Fig. 2b,c). Although the MNP-only sample showed stiffness-dependent signal intensity changes, its MMUS signal was significantly weaker than that of MGVs, leading to lower detection sensitivity of material stiffness (Fig. 2b,c). These results together demonstrate that MGVs embedded in phantoms with lower elastic moduli experience more strain from the same applied magnetic gradient force, resulting in larger vibration amplitudes and stronger ultrasound signals.

To determine whether MGVs can more precisely quantify a wide range of material stiffness values, we used two in vitro phantoms created from two different hydrogel systems with varying stiffness (Fig. 2d and Supplementary Fig. 7). Matrigel-based phantoms were used for softer materials (63 Pa), and agarose gel phantoms were used for materials with elastic moduli ranging from 74 to 5,828 Pa. We observed that the detection range varies with MGV concentration (Fig. 2d,e). The MMUS signal was clearly differentiated in a range of around 63 Pa (SBR = 100.2 ± 31.7) to 564 Pa (10.0 ± 6.9) when using 0.2 nM MGVs, whereas the detection range broadened to 5,828 Pa (24.3 ± 4.9) when using 0.8 nM MGVs (Fig. 2d,e). Furthermore, when very small amounts of MGVs (0.05 nM) were used, the MMUS signal was only visible until 74 Pa, indicating that at this concentration, MGVs are capable of detecting stiffness changes in materials with elastic moduli of less than 74 Pa (Fig. 2d.e). These findings show that depending on the tissue being measured, MGV concentration can be adjusted to generate enhanced or attenuated signals for the acquisition of more accurate MMUS images in diverse tissue types.

**Extended Data Fig. 1:**
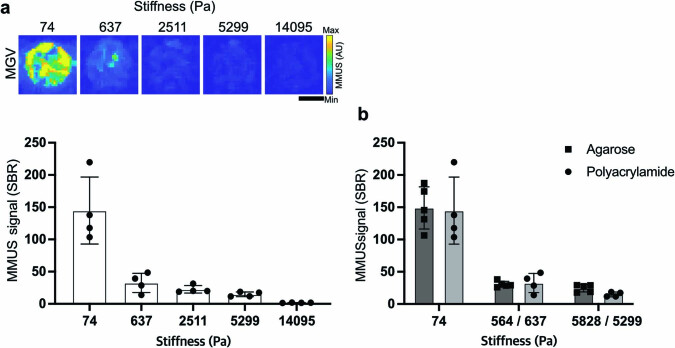
Stiffness-dependent MMUS imaging in a polyacrylamide phantom. (a) MMUS images and SBR quantification of polyacrylamides with stiffnesses ranging from 74 Pa to 14,095 Pa. MMUS imaging was performed using 0.8 nM MGVs (*n* = 4 independent experiments). Min and max on color bars represent 0 and 10000 arbitrary units, respectively. Scale bars, 1 mm. (b) Comparison of the MMUS signal quantification (SBR) in different hydrogels of similar stiffness (*n* = 5 for agarose, *n* = 4 for polyacrylamide). All points represent independent experiments, and line and error bars represent mean ± s.d. Source data

In addition, we imaged MGVs in polyacrylamide gels of various stiffnesses and similar pore sizes (Supplementary Table 1)^12,22,23^, and found an inverse relationship between MMUS signals and stiffness (Extended Data Fig. 1a,b). Furthermore, signal attenuation differences between materials of different stiffnesses were much smaller than the differences in MMUS signals (Supplementary Fig. 8). These results indicate that MMUS imaging can measure stiffness regardless of material composition and pore size (Supplementary Note 1). In vitro cytotoxicity assays showed that treatment with MGVs did not have any significant impact on cellular viability (Supplementary Fig. 9a).

## MGV ability as a stiffness sensor for lung organoid fibrosis

**Extended Data Fig. 2:**
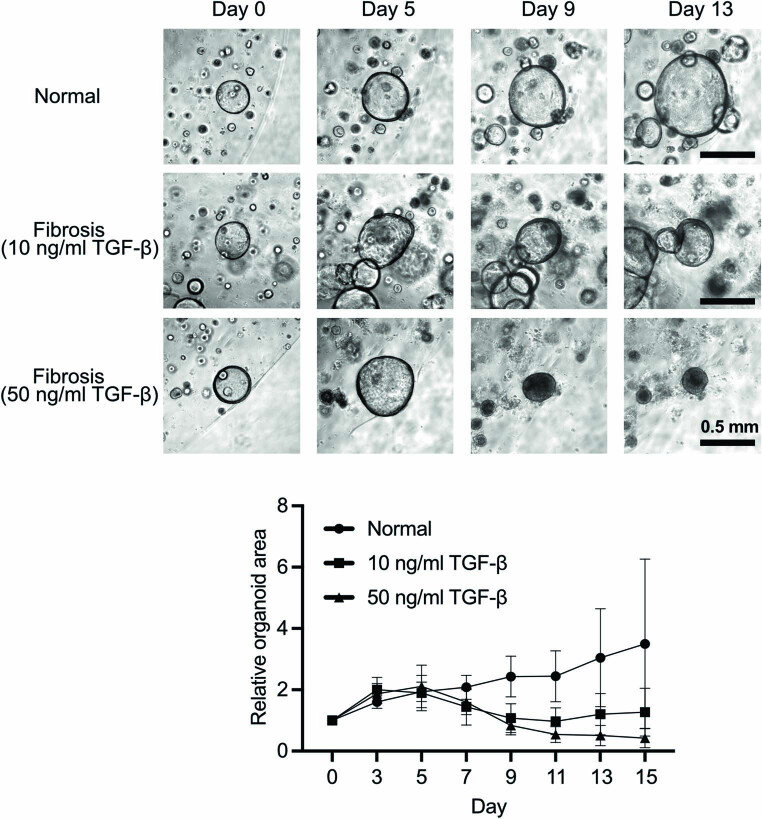
Tracking the fibrotic response of lung organoids during culture. Bright-field images and size quantification graph of lung organoids composed of one normal model and two fibrosis models treated with 10 or 50 ng/mL TGF-β (*n* = 5 biological replicates examined in one experiment). Images were taken from day 0 to day 15 after fibrosis induction. Line and error bars represent mean ± s.d. Source data

Organoids are miniature organ-like constructs and represent a novel in vitro platform for studying disease development^24^. We developed a lung organoid fibrosis model to assess the potential of MGVs for monitoring 3D microenvironment stiffness, aiming to diagnose and observe lung fibrosis progression. Fibrosis was induced in lung organoids using transforming growth factor-β (TGF-β)^25–27^, resulting in decreased organoid size corresponding to fibrosis severity (Extended Data Fig. 2).

**Fig. 3:**
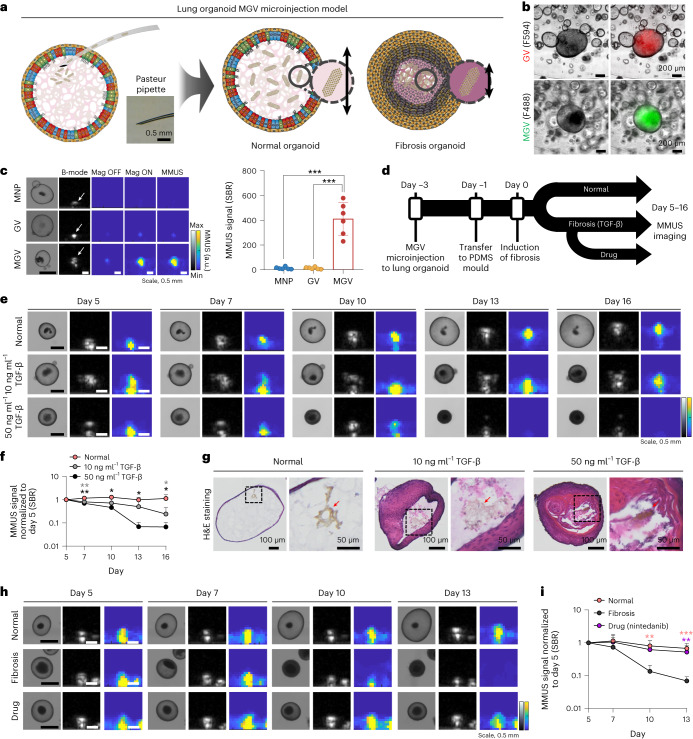
MGV-based MMUS imaging for monitoring fibrosis in a human lung organoid model. **a**, Schematic illustration showing microinjection of MGVs into a lung organoid and detection of stiffness change. **b**, Bright-field and merged fluorescence images of GVs and MGVs in lung organoids. *n* = 2 biological replicates examined in one experiment. **c**, Bright-field, B-mode and MMUS images and quantification of microinjected lung organoids with MNPs, GVs and MGVs. *P* (left–right) = 0.00000061, 0.00000062. *n* = 6 biological replicates examined in one experiment. **d**, Experimental timeline of the preparation of MGV-microinjected lung organoid models and MMUS imaging. **e**,**f**, Bright-field, B-mode, MMUS images (**e**) and SBR quantification (**f**) of MGV-microinjected lung organoids (normal and two fibrosis models). Images were taken from day 5 to day 16 after fibrosis induction. Quantification was conducted using relative MMUS SBR signal normalized to the day 5 value in normal and fibrosis groups. For day 7–16, *P* (grey, left–right) = 0.008486, 0.136217, 0.240127, 0.028171; *P* (black, left–right) = 0.002438, 0.036369, 0.032499, 0.012792. *n* = 3 biological replicates examined in two experiments. **g**, H&E staining of organoid sections in normal and fibrosis groups. Red arrows indicate the localization of MGVs in the organoids. *n* = 1 biological replicate examined in two experiments. **h**,**i**, Bright-field, B-mode, MMUS images (**h**) and SBR quantification (**i**) of MGV-microinjected lung organoids in the normal group, fibrosis group (50 ng ml^−1^ TGF-β) and drug-treated fibrosis group (50 ng ml^−1^ TGF-β + 10 μM nintedanib). Images were taken from day 5 to day 13 after fibrosis induction. Quantification was conducted using relative MMUS SBR signal normalized to the day 5 value in different organoid groups. For day 7–13, *P* (salmon, left–right) = 0.518531, 0.009231, 0.000368; *P* (purple, left–right) = 0.667557, 0.063610, 0.005257. *n* = 7 biological replicates examined in one experiment. Min and max on the parula (MMUS) and grey (B-mode) colour bars represent 0 and 20,000 arbitrary units. All lines and error bars represent mean ± s.d., and significance was determined using one-way ANOVA with Tukey’s multiple-comparisons test in **c**,**f**,**i**: *P < 0.05, **P < 0.01, ***P < 0.001. Source data

**Extended Data Fig. 3:**
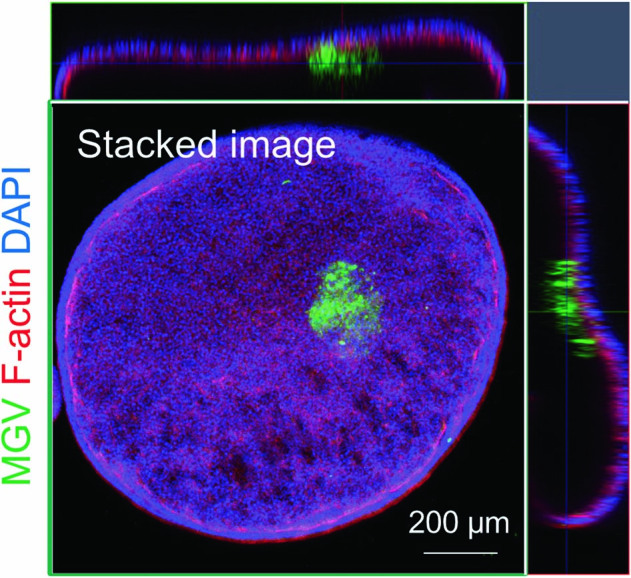
Location of MGV microinjected to lung organoids. Stacked fluorescent image of a lung organoid microinjected with Alexa Fluor 488-conjugated MGVs 19 days after microinjection. F-actin was used for cytoskeleton staining, and DAPI was used for staining nuclei (*n* = 1 biological replicates in two experiments).

To demonstrate the capability of MGV-based MMUS imaging for sensitive detection of fibrosis progression in a lung organoid model, MGVs were microinjected into the lumen of lung organoids, and the difference in MMUS signals was compared between normal and fibrosis organoids (Fig. 3a). Microinjection of MGVs conjugated with fluorescence markers showed that MGVs filled the lumen of lung organoids and remained there for 19 days without leakage (Fig. 3b and Extended Data Fig. 3). Consistent with in vitro experiments, MGVs (SBR = 410.1 ± 132.8) produced 32- and 33-fold enhanced MMUS signals compared with GVs (12.7 ± 8.2) and MNPs (12.1 ± 9.3) in the lumen of lung organoids (Fig. 3c). We hypothesized that fibrotic organoids would exhibit an increased stiffness, which would suppress the magnetically induced movements of MGVs and thus result in weaker MMUS signals (Fig. 3d). Organoids were moved to a polydimethylsiloxane (PDMS) mould 2 days after microinjection, and the next day fibrosis in MGV-injected organoids was induced with TGF-β treatment. MMUS imaging was performed from day 5 to 16 after the induction of fibrosis. The intensity of MMUS signals gradually decreased in the fibrotic lung organoids over a culture period and the decrease in signal was more evident in the organoids treated with 50 ng ml^−1^ TGF-β (Fig. 3e,f). Our results demonstrate that MMUS imaging using MGVs could provide substantial advantages in the detection of fibrosis over other techniques by allowing real-time monitoring of stiffness changes in live lung organoids without fixation.

**Extended Data Fig. 4:**
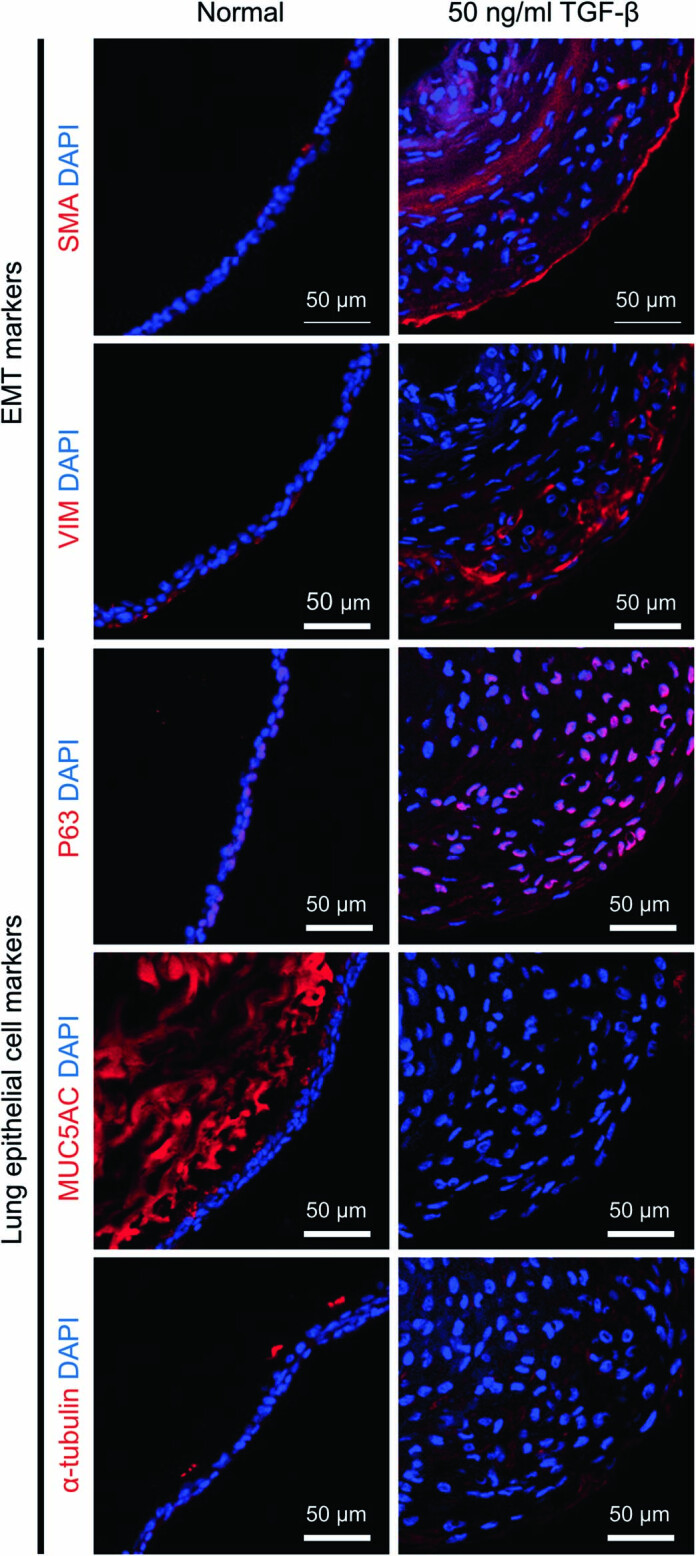
The comparison of protein expression between lung normal organoids and lung fibrosis organoids. Fluorescent images of MGV-microinjected lung organoids stained with EMT markers (VIM and SMA) and lung epithelial cell markers (α-tubulin, MUC5AC, and P63) 16 days after fibrosis induction by treatment with 50 ng/mL TGF-β. DAPI was used for staining nuclei (*n* = 1 biological replicated in on experiments).

Then, the increased fibrosis-induced stiffness in organoids observed by MMUS was validated. Haematoxylin & eosin (H&E) staining revealed thickening of the epithelium layer, abnormal cell growth and MGV localization in the lumen of TGF-β-treated lung organoids in a dose-dependent manner (Fig. 3g). Moreover, in fibrotic lung organoids treated with TGF-β, markers of ciliated (α-tubulin) and goblet (MUC5AC) cells were reduced, while P63-positive basal cells and markers of the epithelial-to-mesenchymal transition (smooth muscle actin (SMA) and vimentin (VIM)) were increased (Extended Data Fig. 4), similar to observations in patients with idiopathic pulmonary fibrosis^28^. Finally, we examined the feasibility of the MGV-bearing lung organoid model for evaluating the efficacy of antifibrosis drugs (Fig. 3h). Nintedanib, well known for its antifibrotic effects on idiopathic pulmonary fibrosis, was tested, and treatment was performed starting from day 5 after fibrosis induction^29^. MMUS signals decreased in organoids with TGF-β-induced fibrosis, but the signals in lung fibrosis organoids treated with drug were maintained at a similar level to that of normal organoids (Fig. 3h,i). MGV imaging in a lung organoid could be used to screen therapeutic drugs for lung fibrosis.

## MGV-based MMUS imaging in a liver organoid fibrosis model

**Fig. 4:**
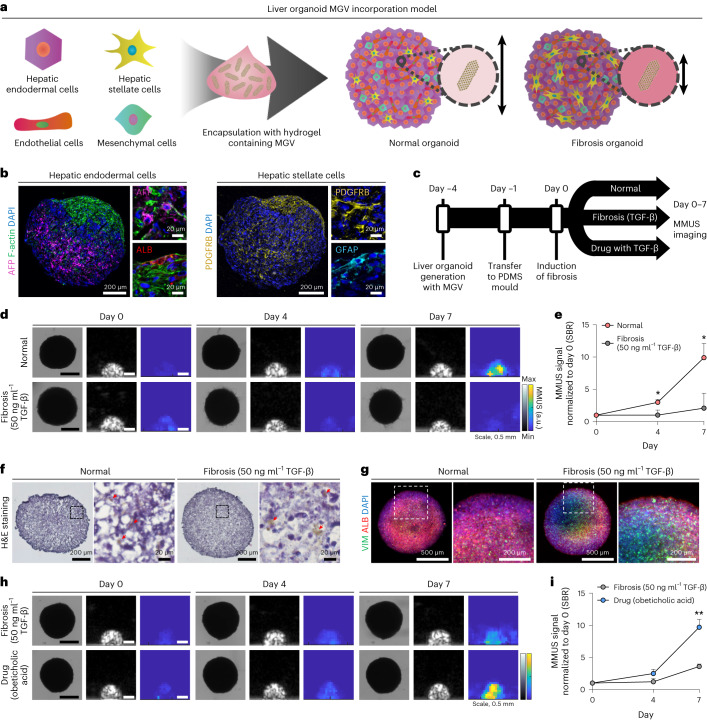
MGV-based MMUS imaging for monitoring fibrosis in human liver organoid models. **a**, Schematic illustration showing MGV encapsulation with four types of cells generating liver organoids and detecting fibrosis-related stiffness changes using MGVs. **b**, Immunofluorescent images showing the expression of hepatic endodermal cell markers (AFP and ALB) and hepatic stellate cell markers (PDGFRB, GFAP) in liver organoids 3 days after organoid generation. TRITC-conjugated phalloidin was used for cytoskeleton (F-actin) staining and DAPI was used for nuclear staining. *n* = 3 biological replicates examined in one experiment. The stained signals are presented in pseudocolour. **c**, Experimental timeline of the preparation of MGV-incorporated liver organoid models and MMUS imaging. **d**,**e** Bright-field, B-mode, MMUS images (**d**) and SBR quantification (**e**) of MGV-incorporated liver organoids (normal and fibrosis model). Images were taken from day 0 to day 7 after fibrosis induction. Quantification was conducted using relative MMUS SBR signal normalized to the day 0 value in normal and fibrosis groups. *P* (day 4–7) = 0.017975, 0.013180. *n* = 3 biological replicates examined in two experiments. **f**, H&E staining of organoid sections in normal and fibrosis groups. Red arrows indicate the localization of MGVs in the organoids. *n* = 2 biological replicates examined in two experiments. **g**, Immunofluorescent images of fibrotic marker (VIM) and mature hepatic marker (ALB) in normal and fibrosis groups. DAPI was used for nuclear staining. *n* = 2 biological replicates examined in two experiments. **h**,**i**, Bright-field, B-mode, MMUS images (**h**) and SBR quantification (**i**) of MGV-incorporated liver organoids in the fibrosis group (50 ng ml^−1^ TGF-β) and drug-treated fibrosis group (50 ng ml^−1^ TGF-β + 10 μM obeticholic acid). Images were taken from day 0 to day 7 after fibrosis induction. Quantification was conducted using relative MMUS SBR signal normalized to the day 0 value in fibrosis and drug-treated fibrosis groups. *P* (day 4–7) = 0.057215, 0.001129. *n* = 3 biological replicates examined in one experiment. Min and max on the parula (MMUS) and grey (B-mode) colour bars represent 0 and 20,000 arbitrary units. All lines and error bars represent mean ± s.d., and significance was determined using an unpaired two-sided *t*-test in **e**,**i**: *P < 0.05, **P < 0.01. Source data

The liver is another important organ for fibrosis modelling, and stiffness is known as an important indicator of the fibrotic liver disease^30^. Accordingly, we tested MGV-based MMUS imaging for detecting the increase in stiffness in a liver fibrosis organoid model. Four types of cells (hepatic endodermal cells, hepatic stellate cells, endothelial cells and mesenchymal cells) were encapsulated in collagen hydrogel containing MGVs, resulting in generation of MGV-incorporated liver organoids (Fig. 4a). This technique allowed localization of MGVs in the extracellular matrix (ECM) in organoids, where the increase in stiffness occurs^31^. Because hepatic stellate cells play an important role in liver fibrosis and collagen is an ECM component highly correlated with increased stiffness in liver fibrosis, our platform contains both cellular and extracellular components suitable for fibrosis modelling. The co-localization of hepatic endodermal cells and hepatic stellate cells in the MGV-incorporated liver organoids suggests that our organoids create a physiologically accurate liver model (Fig. 4b).

**Extended Data Fig. 5:**
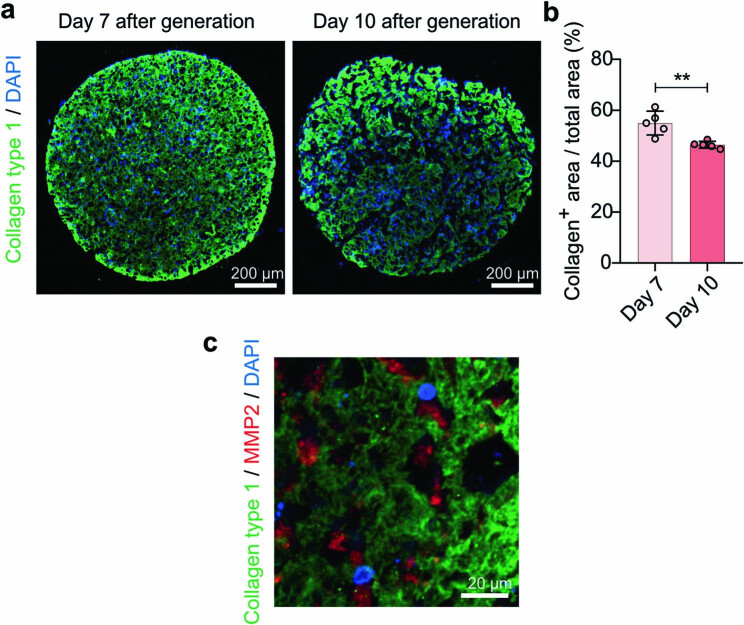
ECM remodeling in liver organoids. (a) Fluorescent images of normal liver organoid sections stained with collagen type 1 on days 7 and 10 after organoid generation. DAPI was used for staining nuclei (*n* = 3 biological replicates in two experiments). (b) Bar graph quantifying the collagen^+^ area / total area (%) of liver organoids on days 7 and 10 after organoid generation (*P* value: 0.004374) (*n* = 5 biological replicates). Line and error bars represent mean ± s.d., and significance was determined using the unpaired two-sided t-test; **: p < 0.01. (c) Fluorescent image of normal liver organoid sections stained with collagen type 1 and MMP2 on day 7 after organoid generation. DAPI was used for staining nuclei (*n* = 3 biological replicates in two experiments). Source data

Induction of fibrosis in liver organoids was done in the same manner as in the lung organoids, and MMUS imaging was performed from 0 to 7 days after induction (Fig. 4c). Although normal and fibrosis liver organoids did not show morphological differences, there was a significant difference in the intensity of the MMUS signals over the culture period (Fig. 4d,e). Interestingly, the MMUS signal intensity increased gradually during the culture of normal liver organoids, indicating a natural decrease in organoid stiffness over time due to active ECM remodelling^32–34^. In normal tissue, ECM homeostasis is regulated by repeated cycles of ECM degradation and synthesis^32,35^. Histological analyses show that the continuous collagen degradation by MMP2 enzymes contributes to the decreased stiffness and increased MMUS intensity of the liver organoids during the culture (Extended Data Fig. 5a–c)^36^. The presence of MGVs in each organoid model was affirmed through H&E staining (Fig. 4f). The upregulation of a fibrotic marker (VIM) and the reduction of a mature hepatic marker (albumin (ALB)) confirmed the induction of fibrosis in liver organoids treated with TGF-β (Fig. 4g). Finally, we examined the applicability of MGV-incorporated liver organoid models to test drugs to treat liver fibrosis. The intensity of MMUS signals in fibrotic liver organoids treated with obeticholic acid (SBR = 9.6 ± 1.2), a drug known to prevent or retard liver fibrosis, was significantly higher than that of the fibrotic organoids without drug treatment (3.5 ± 0.3) (Fig. 4h,i), indicating a notable reduction in stiffness and alleviation of fibrosis by treatment with obeticholic acid. These data demonstrate the possibility of using MGV-incorporated liver organoids as a drug-screening platform for liver fibrosis. The combination of MGV-based MMUS imaging and organoid could also be utilized for other diseases in which a change in stiffness is an important diagnostic indicator, such as acidosis of the brain and cancers^32,33^.

## MGV signal detection in animal liver tissues

**Fig. 5:**
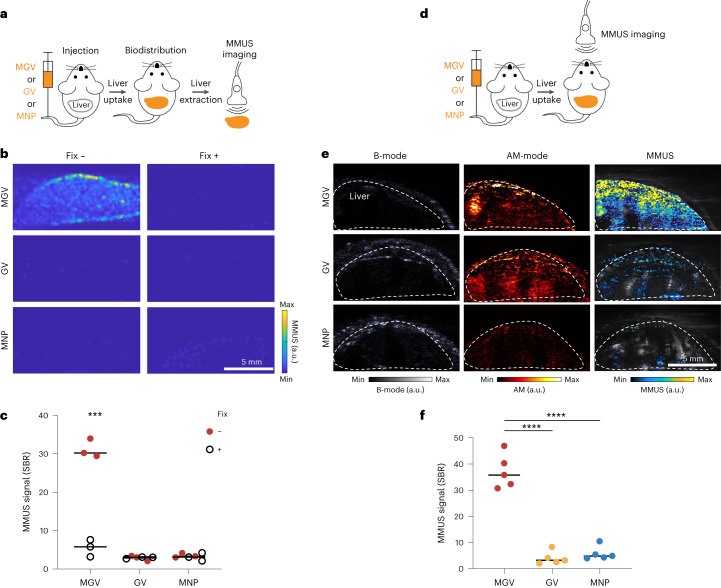
MGV-based MMUS imaging in mouse liver. **a**, Experimental scheme of ex vivo liver MMUS imaging. **b**,**c**, Ex vivo liver MMUS imaging (**b**) and SBR quantification (**c**) before and after fixation. MGVs, GVs or MNPs were injected into the tail vein of mice, and the liver was extracted 5 min after the initial injection. After the first round of imaging, the liver was fixed with 10% formalin for 48 h and imaged again. *P* = 0.000178. *n* = 3 animals per group. **d**, Experimental scheme of in vivo liver MMUS imaging. **e**,**f**, In vivo liver ultrasound images (**e**) and SBR quantification (**f**) of live animals. Three different nanomaterials (MGVs, GVs or MNPs) were injected intravenously and MMUS images were taken after 5 min. B-mode images reveal the position of the liver, AM images show the GV signal inside the liver and Δ MMUS images (parula scale) were overlapped with Doppler images (grey scale) to show the signal below the skin. *P* (left–right) = 0.00000012, 0.00000022. *n* = 5 animals per group. In **b** min and max on the colour bar represent between 0 and 10,000 arbitrary units; in **e** min and max on the parula (MMUS) and hot (AM-mode) colour bars represent between 0 and 500,000 arbitrary units, respectively, and the grey (B-mode) colour bar represent between 0 and 1,000,000 arbitrary units. Each data point represents biologically independent animals, and lines represent the median of each group. Significance was determined using multiple unpaired two-sided *t-*test with Welch’s correction within each group (**c**) and one-way ANOVA with Tukey’s multiple comparisons (**f**): *P < 0.05, **P < 0.01, ***P < 0.001, ****P < 0.0001. Source data

**Extended Data Fig. 6:**
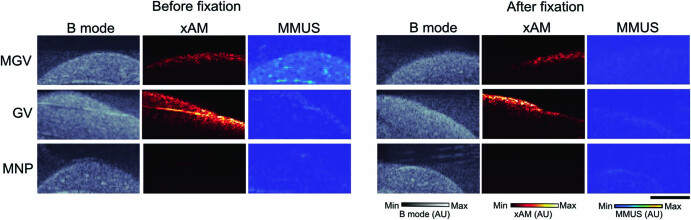
*Ex vivo* liver imaging before and after fixation. Three different groups of nanomaterials (MGVs, GVs, or MNPs) were injected intravenously into different cohorts, and after 5 minutes, the liver was extracted to take ultrasound images. Liver was fixed in 10% formalin for 48 h. B-mode images reveal the shape of the liver, xAM images show the GV signal located at the ultrasound focus, and the Δ images show the MMUS signal (*n* = 3 animals per group). Scale bars, 5 mm. Min and max on color bars for parula (MMUS) and grey (B-mode) colour bar range from 0 to 10000 arbitrary units, and the hot (xAM) colour bar ranges from 0 to 1000000 arbitrary units.

Having demonstrated the ability of MGVs to serve as both MMUS contrast agents and a stiffness sensor in vitro and in cellulo, we tested their capabilities in ex vivo and in vivo animal tissues. To compare their performance as MMUS contrast agents, we performed intravenous injections of MGVs, GVs and MNPs in live mice. To facilitate more specific GV imaging against tissue background, Ana GVs were modified to enhance nonlinear ultrasound contrast under amplitude modulation (AM)^37^. At 5 min post-injection, the liver was removed for ex vivo MMUS imaging (Fig. 5a). The liver was chosen as our model organ because imaging stiffness would be useful for detecting diseases in this organ. In addition, GVs naturally accumulate throughout the liver upon intravenous administration^38,39^. We expected intravenously administered MGVs to be rapidly taken up by the liver, resulting in strong ultrasound contrast in the organ. MGVs were evenly distributed in liver tissue after injection (Supplementary Fig. 10). We observed clear, robust MMUS contrast by MGVs, which exhibited 10-fold stronger signals than those produced by GVs and MNPs (Fig. 5b,c). To demonstrate that MGVs are capable of measuring the mechanical properties of tissues ex vivo, MMUS imaging was done in liver samples fixed with 10% formalin for 48 h^40^. After formalin fixation, the MMUS signals from injected MGVs significantly decreased, corresponding to an increase in tissue stiffness (Fig. 5b,c). The difference in signal attenuation between normal and fixed livers was smaller than that of MMUS signals, indicating that signal differences in MMUS are not primarily influenced by attenuation intrinsic to the tissue (Supplementary Fig. 8). Meanwhile, non-magnetomotive AM ultrasound images showed consistent ultrasound contrast from MGVs before and after fixation, indicating the changes in MMUS signals in the fixed liver are not caused by the collapse or removal of MGVs, but rather by the restricted movement of MGVs (Extended Data Fig. 6). Thus, ex vivo liver imaging demonstrated that increasing stiffness lowered the MMUS signal, similarly as in the organoid model.

**Extended Data Fig. 7:**
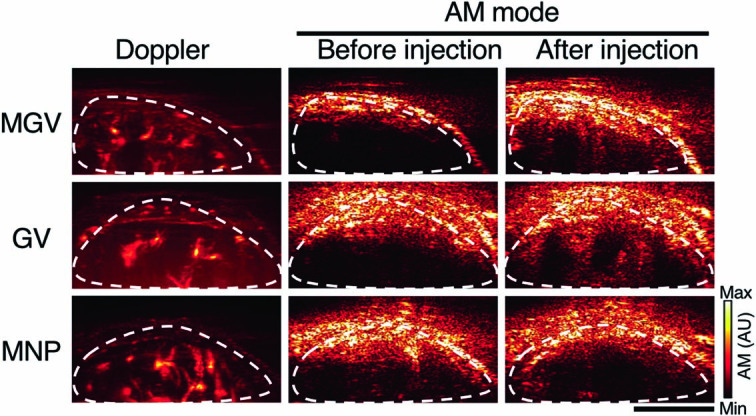
Tracking GV and MGV delivery using AM and Doppler imaging in the liver. Three different groups of nanomaterials (MGVs, GVs, or MNPs) were injected intravenously into different cohorts, and liver AM and Doppler images were taken after 5 minutes. Doppler images reveal the position of the liver, AM images show the signal from GVs or MGVs after injection (*n* = 3 animals per group). Scale bars, 5 mm. Min and max on colour bar ranges from 0 to 1000000 arbitrary units.

In vivo MMUS imaging of live animals is challenging due to skin reflection and breathing artefacts. B-mode and Doppler imaging were used to locate the liver, while ultrafast amplitude modulation (uAM) imaging could visualize robust contrast from MGVs and GVs in the liver (Fig. 5e and Extended Data Fig. 7). After confirming their localization, we set out to test the ability of MGVs to produce robust ultrasound signals that can be visualized in deep tissues by MMUS imaging to image an in vivo biological process within live, breathing animals (Fig. 5d). This is an important challenge in non-invasive, deep-tissue imaging of tissue mechanics as it is well-documented that motion artefacts of live animals reduce accuracy and sensitivity^41^. After intravenous administration, both GVs and MGVs showed enhanced signals in AM images, confirming their delivery into the liver, while MNPs did not produce any detectable signals in vivo. We found that the mean MMUS signal of MGVs (SBR = 37.1 ± 6.5) was 9.3-fold and 6.4-fold stronger than that of GVs (4.0 ± 2.4) and MNPs (5.8 ± 2.6), respectively (Fig. 5e,f). These results demonstrate that MGVs can be used as MMUS contrast agents to improve signal strength and imaging sensitivity in more complex in vivo models.

## Detection of liver fibrosis using MGVs

**Fig. 6:**
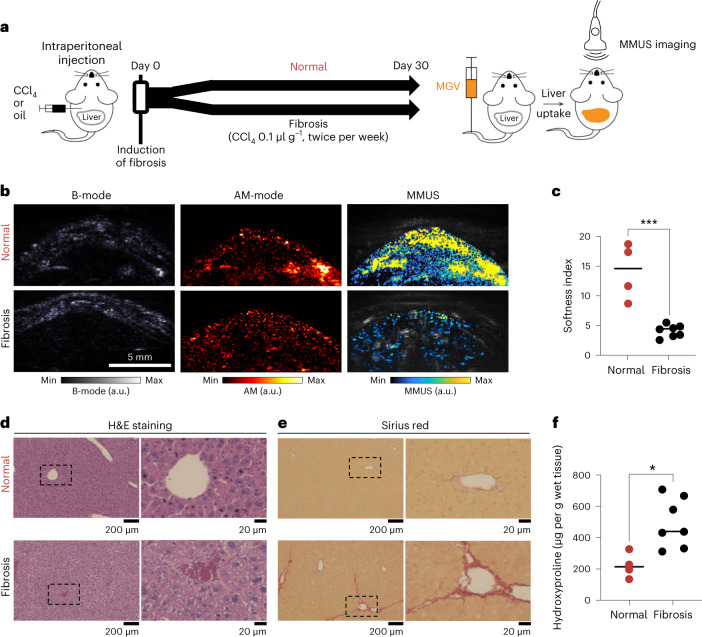
MGV-based MMUS imaging for in vivo fibrosis detection. **a**, Experimental timeline of inducing liver fibrosis in a mouse model. Either CCl_4_ or mineral oil at a volume of 0.1 µl per g (body weight) was injected intraperitoneally twice a week for up to 4 weeks. **b**,**c**, Ultrasound images (**b**) and SBR quantification (**c**) of normal and fibrosis cohorts. The B-mode images reveal the position of the liver, AM images show the GV signal inside the liver, and the Δ MMUS images (parula) were overlapped with Doppler images (grey) to show the signal below the skin. *P* = 0.00033411. *n* = 4 for normal group, *n* = 7 for fibrosis group. **d**, Low-magnification (left) and high-magnification (right) images of H&E-stained sections in two groups. *n* = 3 animals per group. **e**, Low-magnification (left) and high-magnification (right) images of Sirius-red-stained sections in two groups. *n* = 3 animals per grpup. **f**, Quantification of the hydroxyproline content in liver from two groups. *P* = 0.0111. *n* = 4 for normal group, *n* = 7 for fibrosis group. In **b** min and max on the parula (MMUS) and hot (AM-mode) colour bars represent 0 and 500,000, respectively, and the grey (B-mode) colour bar ranges from 0 to 1,000,000 arbitrary units. Each data point represents biologically independent animals, and lines represent the median of each group. Significance was determined using an unpaired two-sided *t*-test: *P < 0.05, **P < 0.01, ***P < 0.001, ****P < 0.0001. Source data

After establishing MGVs as excellent contrast agents for in vivo MMUS imaging, we then investigated whether our MGV-based system could function as a stiffness sensor to diagnose in vivo disease models. To induce liver fibrosis in mice, we injected CCl_4_, which increases hepatic stiffness during the progression of fibrosis for 30 days post-injection^42^. After 4 weeks of CCl_4_ treatment, MMUS imaging was performed to assess the mechanical properties of fibrotic and normal livers (Fig. 6a). Intravenously administered MGVs were taken up by liver tissues and retained their ultrasound scattering property, as evidenced by robust ultrasound contrast under AM. Although AM imaging confirmed that similar quantities of MGVs were delivered to the livers of both control and fibrotic mice, we observed a substantial reduction in MMUS signal in the fibrosis-induced cohort (Fig. 6b). A softness index, which we used as a quantitative indicator of in vivo tissue stiffness based on MMUS and AM imaging, was significantly lower in the fibrosis group (4.2 ± 1.0) than in normal controls (14.0 ± 4.6) (Fig. 6c), consistent with previous observations that liver stiffness increases with the progression of fibrosis^43^. Histological and biochemical analyses confirmed the induction of liver fibrosis, as evidenced by pronounced morphological alteration, disruption of tissue architecture, fibre extension and increased collagen accumulation (Fig. 6d–f and Supplementary Fig. 11). No signs of fibrosis, inflammation and immune response were observed in animals injected with MGVs, suggesting these vesicles have high biocompatibility and negligible immunogenicity (Fig. 6d–f and Supplementary Fig. 9b,c). By utilizing clinically relevant ultrasound frequencies (6.25 MHz) and biocompatible superparamagnetic iron oxide nanoparticles, we could also demonstrate the clinical potential of our MGV-based system (Supplementary Figs. 12 and 13). Moreover, organoid and liver tissue modulus could be estimated by correlating MGV concentration with B-mode and MMUS signals to make our system comparable across models (Supplementary Fig. 14a–e and Supplementary Note 2). These results demonstrate the potential of MGVs to serve as contrast agents for non-invasive detection of mechanical changes in vivo.

## Outlook

Our results establish a new class of hybrid protein nanostructures (MGVs) as nanomaterial-based, magneto-acoustically modulated MMUS contrast agents for non-invasive and sensitive imaging and measurement of tissue elasticity in vivo. The clinical potential of MGV-based MMUS imaging for ultrasound-imaging-based diagnosis and drug screening of a wide range of diseases, including fibrosis, has been demonstrated. Our MGV-based MMUS imaging technique provides several advantages over microbubble-based monitoring and other techniques, such as MRI, in disease monitoring^44–47^. It offers wider availability, lower cost and improved stability and tissue accessibility, making it suitable for cost-effective and long-term disease monitoring. Improvements in magnetic field gradients, quantifiability and integration with existing strongly magnetic instruments such as MRI or magnetic particle imaging should make it possible to enhance the widespread use of MGV-based imaging. Moreover, the use of MGVs in stem-cell-derived organoid systems will offer a valuable tool for investigating mechanical properties and enable tissue-mechanics-based diagnosis and prediction of therapeutic outcomes in human diseases.

## Methods

All experiments were performed in accordance with relevant guidelines and ethical regulations that were approved. The use of human lung tissues for lung organoid generation was approved by the Institutional Review Board (IRB) of Severance Hospital (IRB number 4-2021-1555). Tissue fragments were collected from patients undergoing lung surgery after obtaining their informed consent. A human-induced pluripotent stem cell (hiPSC) line (CHO) was kindly provided by the Yonsei University School of Medicine, and the use of hiPSCs for the liver organoid study was approved by the IRB of Yonsei University (permit numbers 7001988-202104-BR-1174-01E, 7001988-202104-BR-1175-01E). All in vivo experiments were conducted in accordance with protocol 1735 approved by the California Institute of Technology’s Institutional Animal Care and Use Committee.

### Preparation of MNPs

Zinc-doped iron oxide (Zn_0.4_Fe_2.6_O_4_) nanoparticles were synthesized by previously published procedures^20^. To synthesize zinc-doped iron oxide (Zn_0.4_Fe_2.6_O_4_) nanoparticles, 0.6 g of zinc(II) chloride (ZnCl_2_, ≥98%, Sigma-Aldrich) and 1.756 g iron(III) acetylacetonate (Fe(acac)_3_, ≥98%, Sigma-Aldrich) were placed in a three-neck round-bottom flask in the presence of 5 ml of oleic acid (Sigma-Aldrich), 20 ml of oleylamine (Sigma-Aldrich) and 20 ml of trioctylamine (98%, Sigma-Aldrich) under argon gas. After the synthesis, the precipitation and washing were performed using ethanol and toluene. Silica coating was used to make these nanoparticles water soluble and functionalizable. To begin, for 1 mg of MNPs the surface was treated with 10 µl of tetraethyl orthosilicate (Sigma-Aldrich), 12.6 ml of cyclohexane (Deajung), 786 mg of IGEPAL CO-520 (Sigma-Aldrich) and 105 µl of ammonium hydroxide solution (28%, Sigma-Aldrich) for 24 h at room temperature. The second layer was coated with 6 µl of 3-aminopropyl trimethoxysilane (Sigma-AldrichA) for 2 h. After separation with tetramethyl ammonium hydroxide (TMAOH, 97%, Sigma-Aldrich), to introduce azide groups on the surface of the nanoparticles, silica-coated nanoparticles (1 mg) were then coated with m-dPEG12-TFP ester (9 mg, Quanta BioDesign), azido-dPEG12-TFP ester (1 mg, Quanta BioDesign) in dimethylsulfoxide for 2 h at room temperature. Nanoparticles were isolated using a MidiMACS separator column and were dispersed in 10 mM phosphate buffer.

### Preparation of GVs

*Anabaena* gas vesicles were obtained according to published procedures^48^. GVs were isolated from *A. flos-aquae* using hypertonic lysis and purified using centrifugally assisted flotation. Stripped GVs were prepared by treatment with 6 M urea solution followed by an additional centrifugally assisted flotation and removal of the subnatant. To functionalize DBCO on the surface of the GVs, DBCO-sulfo-NHS ester (Click Chemistry Tool) was mixed with GVs at a molar ratio of 1:10 in deionized water (DIW) for 4 h at 4 °C at 30 r.p.m. in a vertical shaker. Functionalized GVs were dialysed in DIW for 72 h with a water exchange every 24 h.

### Development and characterization of MGVs

MGVs were developed by conjugating MNPs to GVs at a molar ratio of 1:100 for 4 h at 4 °C at 30 r.p.m. in a vertical shaker. After 4 h, the MGVs were purified three times using buoyancy purification at 300*g*, 4 °C, 24 h, and the solvent was replaced with phosphate-buffered saline (PBS) each time. We characterized the morphology, size and magnetic susceptibility of MGVs by transmission electron microscopy (TEM, JEOL 2100, DigitalMicrograph 3.22.1461.0, JEOL), dynamic light scattering (Zetasizer Nano ZS, software 7.12) and vibrating-sample magnetometry (Vibration 7407-S, software 4.9.0, Kake Shore Cryotronics), respectively. ICPMS (ICAP 7200 Duo + ASX-560, Qtegra 2.6.2270, Thermo Fisher Scientific) was used to determine the concentration of MNPs in MGVs. The concentrations of iron and zinc ions measured by ICPMS were converted to numbers of MNPs. The concentration of GVs was then calculated by dividing 186 by the average number of MNPs attached to GVs as determined by our TEM images, which was manually calculated (Supplementary Fig. 3).

### Experimental MMUS imaging set-up

#### Magnet set-up

A schematic illustration of our custom-built MMUS system is illustrated in Fig. 1a. A multipurpose data-acquisition module (USB6003, National instruments), power supply (RSP-1000-24, 24 V, 40 A, Meanwell) and solid-state module (SSR-40DD, FOTEK) were commercially available items. To meet the imaging system requirements, the ultrasound system was modified to output a trigger signal prior to imaging to generate a magnetic field. The magnetic field pulse and strength were controlled by using a customized LabVIEW system. The field generator was connected to a coil consisting of multiple turns with a magnetic coil. To increase the magnetic flux density and to localize the magnetic field in the centre of the coil, ferritic stainless steel was embedded. The core size was 5 mm in diameter and 100 mm in height. To better focus the field onto a smaller region of interest, a symmetric conic frustum was cut at 56° on the top side. The magnetic pulse strength, measured at 6 mm above the iron-core tip using a digital gaussmeter (DSP 475, Lakeshore Inc., Westerville, OH), was 0.03 T, which was used for the imaging experiments. Different magnetic strengths were achieved by adjusting the distance between the sample and the magnet.

#### MMUS imaging and processing

For MMUS imaging, the phantoms were submerged in PBS, and ultrasound images were acquired using a Verasonics Vantage programmable ultrasound scanning system with an L22-14v 128-element linear array transducer with a 0.10 mm pitch, an 8 mm elevation focus, a 1.5 mm elevation aperture and a centre frequency of 15.6 MHz with 67% −6 dB bandwidth (Verasonics). Measured peak voltages received by the transducer were collected as I/Q data. Two sets of ultrasound I/Q data were collected for in vitro and organoid imaging at each loop containing a pulse sequence consisting of five tilted plane waves (varying from −6° to 6°), each containing 500 ensemble coherently compounded frames, collected at a frame rate of 500 Hz with a voltage of 3 V. A total of 20 loops of images were collected per set. The first set was taken as a background frame for background subtraction with the magnetic field off (Mag OFF). The second set was taken with the magnetic field on (Mag ON), during which the function generator was triggered for 2,000 µs prior to the beginning of the imaging.

To obtain each image, I/Q data were processed with quadrature detection used to extract the generated movement based on the excitation frequency^49^. Briefly, for a set of *N* frames, let *R*_I_(*x*,*y*,*n*) + *j*R_Q_(*x*,*y*,*n*) represent an element in this I/Q array with *n* running from 1 to *N*, and *R*_I_ and *R*_Q_ representing the in-phase and quadrature signal, respectively. First, the received I/Q data were phase unwrapped to generate a new 3D array *r*_unwrapped_(*x*,*y*,*n*) = arg(*R*_I_(*x*,*y*,*n*) + *j*R_Q_(*x*,*y*,*n*)). Then, quadrature detection was used to tease out the signal that oscillates at the magnetic pulse frequency (*f*_0_)

$$R\left(x,y,n\right)=r_{{\rm{unwrapped}}}\left(x,y,n\right)\times {\mathrm{e}}^{\,{j\times 2\uppi\times {f}}_{0}\times {n}\times {\delta }{t}}.$$

To calculate the displacement amplitude at frequency *f*_0_ for each pixel, all the frames were averaged to calculate $\underline{R(x,y)}$ for the quadrature-detected sequence *R*(*x*,*y*,*n*). The mean value was used rather than a low-pass filter to determine the displacement amplitude at *f*_0_, which was obtained as

$$A(x,y)=2|\underline{{R}(x,y)}|=2\sqrt{{I}{(x,y)}^{2}+{Q}{(x,y)}^{2}}.$$

Finally, ultrasound Δ images were constructed by subtracting the Mag OFF frame from the Mag ON frame. Regions of interest (ROIs) were defined to capture the ultrasound signal from the phantom well or organoid region on various images such as MMUS (Δ), B-mode or cross-propagating amplitude modulation (xAM) images. All in vitro phantom experiments had the same ROI dimensions. For organoid models, ROIs were selected in B-mode images in which the organoid size was not same in all cases. Background ROIs were chosen in areas where no sample was present. The mean pixel intensity was calculated for each ROI, and the signal from the background region and the sample region was calculated as the SBR.

### Ultrasound phantom preparation

#### In vitro phantom

To produce in vitro MMUS imaging phantoms, wells were cast with molten 0.5% (w/v) agarose in PBS using a custom 3D-printed template^48^. MGV, MNP or GV (DBCO-functionalized GV) samples were mixed 1:1 with 50 °C agarose and injected into wells prior to solidification. Matrigel was stored at 4 °C until loaded and solidified for 30 min at 37 °C. Agarose or Matrigel hydrogels and samples were made at a concentration two times greater than the final required concentration. For polyacrylamide gels, the desired concentrations of acrylamide and bis-acrylamide were combined with 0.002 M lithium phenyl-2,4,6-trimethylbenzoylphosphinate solution dissolved in DIW (Supplementary Table 1). After the combination, these gels were cast in a custom 3D-printed template. The MGV concentration was 20 times greater than the final required concentration and was mixed 1:20 with polyacrylamide solutions before being cast into each well. The gels were solidified for 5 min with an ultraviolet lamp (DR-301C, MelodySusie).

#### Organoid phantom

For MMUS imaging of organoids, a PDMS mould was fabricated. PDMS solution was prepared by mixing PDMS prepolymer (Sylgard 184; Dow Corning) and curing agent (Dow Corning) at a ratio of 10:1 (v/v). Then the mixture was poured into 60 mm Petri dishes and cured in a drying oven for 4 h after removing bubbles using a vacuum chamber. The centre of the cured PDMS mould was punched to make chambers for the organoids. After sterilizing each PDMS mould with ultraviolet irradiation for 30 min, MGV-microinjected lung organoids or MGV-incorporated liver organoids were encapsulated in growth-factor-reduced Matrigel (Corning) and transferred to the chambers in the mould. After the gelation of Matrigel, the organoids were cultured in growth medium, and MMUS imaging was performed after replacing the medium with 1× PBS (Sigma-Aldrich). For fibrosis induction, lung organoids were cultured in medium including recombinant human TGF-β1 (Peprotech) without A83-01 (Tocris). For the drug tests with lung organoids, 10 μM nintedanib (Sigma-Aldrich) was administered to the organoids every 2–3 days starting from day 5 after fibrosis induction. Liver organoids were also cultured in medium containing TGF-β1 for fibrosis induction, and 10 μM obeticholic acid (Selleck) used for the drug tests was administered to the organoids every 2–3 days starting from the first day of fibrosis induction (day 0).

### Generation of MGV organoids

Details of how the MGV-microinjected lung and MGV-incorporated liver organoids are generated are given in the Supplementary Information. Information on the maintenance and immunostaining of organoids is also given in the Supplementary Information. Chemicals for synthetic operations were purchased from common suppliers (Sigma-Aldrich, Thermo Fisher Scientific, Abcam, etc.) and were stored at the suitable temperatures.

### Animal experiments

Animals were randomly assigned to experimental groups by the animal facilities. Animals were housed in a facility maintained at 71–75 °F and 30–70% humidity, with a lighting cycle of 13 h on and 11 h off (light cycle 6:00–19:00). Throughout all injection and imaging procedures, mice were anaesthetized with ~1–2.5% isoflurane. Mice were positioned with the liver facing directly upwards. Prior to each experiment, ultrasound gel was centrifuged at 2,000*g* for 10 min to remove bubbles, heated to 37 °C and then carefully applied to the bodies of the mice. To obtain a precise signal, for all ex vivo and in vivo models, stripped GVs were used. Stripped MGVs were prepared in the same manner as MGVs. The concentration of GVs was matched to the concentration of MGVs. MNP concentration was also matched to the MNP concentration found in the MGVs.

#### Ex vivo imaging

For ex vivo imaging, three C57 male mice aged 8 weeks were injected intravenously in the tail vein with 2,280 pM (OD_500_ = 20) of MGVs and were killed 5 min later. The concentration used for the injections was chosen based on previous research. After the animals had been killed, their livers were harvested for ex vivo imaging. For MMUS imaging, the liver was cast in 0.5% (w/v) agarose in a 100 mm Petri dish and solidified for 10 min. After the first series of imaging, the tissue was fixed for 48 h in 10% formalin at 4 °C. The second series of ex vivo imaging occurred after fixation.

#### Live animal imaging

Five 4-week-old C57 male mice were intravenously injected with MGVs for in vivo imaging. The regions of interest were positioned in the liver tissue using B-mode and Doppler anatomical imaging. The concentration used for injections was chosen based on previous research. MMUS imaging was performed before and after the injection of MGVs with the magnetic field on. MGVs (100 µl at 2,280 pM (OD_500_ = 20)) were injected intravenously via the tail vein, and MMUS images were taken 5 min post-injection.

#### Fibrosis model

Prior to the in vivo fibrosis experiment, animals were randomized between experimental groups; blinding was not necessary. Four-week-old C57 male mice were treated with CCl_4_ (1 µl per g (body weight), 1:4 dilution with mineral oil, *n* = 7) or with mineral oil alone (1 µl per g (body weight), *n* = 4) via intraperitoneal injection two times per week for 4 weeks^50,51^. After 4 weeks, the ROIs were positioned in the liver tissue using B-mode and Doppler imaging. MMUS imaging was performed before and 5 min after injection with the magnetic field on. MGVs (2,280 pM) were injected intravenously via the tail vein in the normal and fibrosis model groups. After MMUS imaging, livers were harvested. Fresh tissue was homogenized and used for a hydroxyproline assay (Sigma-Aldrich). Other parts of the tissue were fixed for 24 h in 10% formalin and then submerged in 70% ethanol for storage. Next, the fixed tissue was embedded in paraffin, sectioned and stained with H&E and Sirius red (Abcam). The images were examined under a laser scanning confocal microscope (Zeiss LSM880, Jena, Germany) and analysed using Zen v.3.0 (Zeiss).

#### In vivo ultrasound imaging

We employed a recently developed method of uAM to precisely visualize and quantify ultrasound contrast in vivo^38^. Due to the attenuation of applied sound waves caused by the body, we increased the sound pressure to 370 kPa with the same Verasonics system using an L22-14v transducer, which did not collapse either MGVs or GVs^38^. For each loop, the data were collected at a frame rate of 350 Hz with a voltage of 6 V (370 kPa). The pulse sequence consisted of four bursts repeated at three different amplitudes with four different polarity patterns (varying from −14° to 14°). Each burst contained 500 ensemble coherently compounded frames. Two sets of images were taken prior to and following injection. The first set was used as a baseline for background subtraction purposes, with the magnetic field activated prior to injection (before). The second set was taken 5 min after injection with the magnetic field activated (after), with the function generator triggered 2,000 µs prior to the start of imaging. A total of 20 looped images were collected per set. We removed frames with poor breathing artefacts based on their Doppler images. To obtain in vivo ultrasound images, the same processing procedures were used as in vitro ultrasound imaging, and ultimately, ultrasound Δ images were constructed by subtracting the after image from the before image. For in vivo MMUS signal quantification, the Δ images were used. ROIs were selected consistently to exclude edge effects from the skin. Background ROIs was selected where there was no sample at all. The mean pixel intensity was calculated for each ROI, and the signal from the background region and sample region was calculated as the SBR. For the fibrosis experiments, the ratio of the MMUS (SBR) signal to the uAM (SBR) signal was calculated and reported as a softness index.

### Statistics and reproducibility

All data are presented as box plots or line plots expressed as mean ± s.d. unless otherwise indicated. The number of experiments and statistical comparisons are specified for each experiment and reported in the figure legends. Sample sizes were chosen on the basis of preliminary experiments to have sufficient replicates for statistical comparison. Data distribution was assumed to be normal but this was not formally tested. Statistical calculations were performed in GraphPad Prism 9. The microscopic and ultrasound images in the figures are representative images obtained from independent samples, biological replicates or biologically independent animals with similar results. No data were excluded from the analyses. The investigators were not blinded to allocation during experiments and outcome assessment unless otherwise indicated. For cell culture experiments, standardized cell culture conditions and samples used in each set of experiments were equal to minimize variation across samples, except the experimental condition being tested. Cultured lung and liver organoids were randomly assigned for each group when they reached each specified time point. For mouse experiments, animals were randomly assigned to experimental groups by the animal facilities and cages of animals were randomly chosen for the experimental groups versus control conditions. In all other experiments, samples were allocated randomly and performed with appropriate control.

### Reporting summary

Further information on research design is available in the Nature Portfolio Reporting Summary linked to this article.

## Online content

Any methods, additional references, Nature Portfolio reporting summaries, source data, extended data, supplementary information, acknowledgements, peer review information; details of author contributions and competing interests; and statements of data and code availability are available at 10.1038/s41563-023-01688-w.

### Supplementary information

Supplementary Information Supplementary Methods, Notes 1–3, Figs. 1–14, Table 1 and References.

Reporting Summary

### Source data

Source Data Fig. 1 Statistical source data for Fig. 1.

Source Data Fig. 2 Statistical source data for Fig. 2.

Source Data Fig. 3 Statistical source data for Fig. 3.

Source Data Fig. 4 Statistical source data for Fig. 4.

Source Data Fig. 5 Statistical source data for Fig. 5.

Source Data Fig. 6 Statistical source data for Fig. 6.

Source Data Extended Data Fig. 1 Statistical source data for Extended Data Fig. 1.

Source Data Extended Data Fig. 2 Statistical source data for Extended Data Fig. 2.

Source Data Extended Data Fig. 5 Statistical source data for Extended Data Fig. 5.

## Data Availability

The data presented in this study are available in the Source data. Additional information and requests for resources and reagents that support the findings of this study are available from the corresponding author upon reasonable request. Source data are provided with this paper.

## Code availability

Ultrasound data acquisition and analysis code is available on the Shapiro laboratory GitHub at https://github.com/shapiro-lab.
