# Supplementary material for: Magneto-acoustic protein nanostructures for non-invasive imaging of tissue mechanics in vivo
Source: Nat Mater. 2023 Oct 16;23(2):290–300. doi: 10.1038/s41563-023-01688-w (PMC10837075; doi:10.1038/s41563-023-01688-w)
Supplement: Supplementary file 1 — Supplementary Methods, Notes 1–3, Figs. 1–14, Table 1 and References. [file 41563_2023_1688_MOESM1_ESM.pdf]

nature materials

Article https://doi.org/10.1038/s41563-023-01688-w

# Magneto-acoustic protein nanostructures for non-invasive imaging of tissue mechanics in vivo

In the format provided by the authors and unedited

# Supplementary Information


1. **Supplementary Methods**
2. **Supplementary Notes 1-3**
3. **Supplementary Figures**
4. **Supplementary Table**
5. **Supplementary References**

# Supplementary Methods

## Rheometer measurement

To prepare the hydrogel samples for the rheometer, agarose or Matrigel was solidified at 25 °C or 37 °C, respectively, at the desired concentration. The rheometer (Anton Paar MCR102, version V1.24.549, Anton Paar) was set to frequency sweep mode from 100 Hz–0.1 Hz (logarithmic, 16 points) and the (ocillatory) shear strain was set to 1%. Elastic modulus values were determined using the storage modulus obtained at 1 Hz.

## Organoid experiments

**Generation of lung organoids and MGV microinjection.** Lung organoids were prepared from human lung tissues harvested with the patients' consent. The use of human lung tissues for lung organoid generation was approved by the Institutional Review Board (IRB) of Severance Hospital (IRB No: 4-2021-1555). Tissue fragments were collected from patients undergoing lung surgery after acquiring their informed consents. The basal medium used to isolate lung cells from tissues and to prepare growth medium for lung organoid culture consisted of advanced DMEM/F12 (Dulbecco's Modified Eagle Medium/Ham's F-12) containing 2 mM GlutaMax, 10 mM HEPES (N-2-hydroxyethylpiperazine-N-2-ethane sulfonic acid) and 1% (v/v) penicillin/streptomycin (P/S) (all from Thermo Fisher Scientific, Waltham, MA, USA). Then, organoid growth medium was prepared using the basal medium supplemented with 10% (v/v) R-spondin1-conditioned medium, 1× B27 (Thermo Fisher Scientific), 100 ng/mL mouse Noggin (Peprotech), 100 ng/mL human fibroblast growth factor-10 (FGF10, Peprotech), 25 ng/mL human fibroblast growth factor-7 (FGF7, Peprotech), 1.25 mM N-acetyl cysteine (Sigma-Aldrich), 5 mM nicotinamide (Sigma-Aldrich), 500 nM A83-01 (Tocris), and 100 µg/mL primocin (InvivoGen, San Diego, CA, USA). R-spondin1-conditioned medium was prepared using HEK293T cells expressing Rspo1-Fc, which were obtained from Calvin Kuo's laboratory at Stanford University. Lung organoids were generated using a previously reported protocol[1]. Briefly, harvested human lung tissues were fragmented using scissors, and then washed three times with Dulbecco's phosphate buffered saline (DPBS, Sigma-Aldrich). The tissues were incubated in collagenase type II (2 mg/mL solution in basal medium, Thermo Fisher Scientific) with gentle shaking for 1 h at 37 °C. Digested tissues were then washed with DPBS and strongly pipetted with 0.1% (v/v) fetal bovine serum (FBS, Thermo Fisher Scientific) diluted in DPBS to separate cells from the tissue. The suspension was filtered with a 70 µm strainer to remove the tissue debris, and the number of isolated cells was counted. Then, cells were encapsulated in growth factor-reduced Matrigel at a concentration of $5 \times 10^6$ cells/mL and were moved into a 48 well plate. After gelation for 10 minutes, growth medium was added to each well. The growth medium was replaced every 3-4 days, and 10 µM Y27632 (BioGems International, Inc., Westlake Village, CA, USA) was added to the culture for the first 4 days. The microinjections of MGVs were performed using a method similar to microinjection into embryos in a previous study[2]. The end of a Pasteur pipette was made narrow for microinjection using an alcohol lamp, and it was connected to a mouthpiece and rubber tubing. MGV solution (OD16) was microinjected into lung organoids, and the organoids were then cultured for 2 days to allow for stabilization. After Matrigel encapsulating the organoids was degraded by treatment with cell recovery solution (Corning), the harvested MGV-microinjected lung organoids were transferred to the organoid

chamber of a PDMS mold for MMUS imaging. Microinjection of GVs or MNPs was performed in the same manner as microinjection of MGVs. Microinjected organoids were imaged using a microscope (IX71, Olympus, Tokyo, Japan) and analyzed using ocular sofware (version 2.0, olympus life science) for Figure 3 and Extended figure 2. The organoid microscopic images for the time point experiment were imaged and analyzed using the EVOS® FL Auto Cell Imaging system (software v.16, Thermo Fisher Scientific).

**Human iPSC maintenance**. A human induced pluripotent stem cell (hiPSC) line (CHO) was kindly provided by the Yonsei University School of Medicine, and the use of hiPSCs for the liver organoid study was approved by the Institutional Review Board (IRB) of Yonsei University (permit number: 7001988-202104-BR-1174-01E, 7001988-202104-BR-1175-01E). The hiPSC line was characterized for pluripotency and regularly checked for mycoplasma contamination. Culture of hiPSCs was performed using StemMACS iPSC-Brew XF medium (Miltenyi Biotec, Bergisch Gladbach, Germany) under Matrigel-coated, feeder-free conditions. Cells were passaged every 5-7 days using ReLeSR (STEMCELL Technologies, Vancouver, BC, Canada) and maintained within passages 20 to 50.

**Generation of MGV-incorporated human liver organoids.** For generation of liver organoids, four types of cells (hepatic endodermal cells, hepatic stellate cells, endothelial cells, and mesenchymal cells) were prepared. Hepatic endodermal cells and hepatic stellate cells were differentiated from hiPSCs. Human umbilical vein endothelial cells (HUVECs) and human mesenchymal stem cells (hMSCs) were purchased from Lonza (Basel, Switzerland). The differentiation protocol for hepatic endodermal cells was adapted from the previous methods with slight modifications[3,4]. Briefly, hiPSCs were seeded on Matrigel-coated 6-well plates with mTeSR plus medium (STEMCELL Technologies) and 10 μM Y27632. After 24 h, the medium was changed to RPMI 1640 (Thermo Fisher Scientific) and supplemented with 100 ng/mL activin A (R&D Systems, Minneapolis, MN, USA) and 1% B27 minus insulin (Thermo Fisher Scientific), and the cells were cultured for 4 days to induce definitive endodermal cells. CHIR99021 (3 μM, LC laboratory, Woburn, MA, USA) was added to the medium only on the first day of the 4-day differentiation protocol. Then, the cells were further cultured for an additonal 3 days to induce hepatic endodermal cells in RPMI 1640 medium supplemented with 1% B27 (Thermo Fisher Scientific), 20 ng/mL bone morphogenetic protein 4 (BMP4, R&D Systems), and 10 ng/mL fibroblast growth factor-basic (bFGF, Peprotech). The differentiation protocol for hepatic stellate cells was adapted from a previously reported method with slight modifications[5]. Briefly, hiPSCs were seeded on Matrigel-coated 6 well plates with mTeSR Plus medium and 10 μM Y27632. After 24 h, cells were treated with 20 ng/mL BMP4 in a hepatic stellate cell basal medium composed of 40% (v/v) MCDB201 (Sigma-Aldrich), 57% (v/v) DMEM low-glucose (Thermo Fisher Scientific), 1% (v/v) P/S, 0.1 mM β-mercaptoethanol (Sigma-Aldrich), 100 μM L-ascorbic acid (Sigma-Aldrich), 2.5 μM dexamethasone (Sigma-Aldrich), 0.25× insulin-transferrin-selenium supplement (Sigma-Aldrich), and 0.25× linoleic acid (Sigma-Aldrich) for 4 days to induce mesodermal progenitors. Cells were further differentiated into mesothelial cells for an additional 4 days. Cells were treated with 20 ng/mL BMP4, 20 ng/mL fibroblast growth factor-acidic (FGF1, Peprotech), and 20 ng/mL fibroblast growth factor-3 (FGF3, R&D Systems) for the first 2 days and with 20 ng/mL FGF1, 20 ng/mL FGF3, 5 μM retinol (Sigma-Aldrich), and 100 μM palmitic acid (Sigma-Aldrich) for the remaining 2 days. For the final stage of hepatic stellate cell differentiation, 5 μM retinol and 100 μM palmitic acid were added to the hepatic stellate cell basal medium for 4 additional days. The growth medium was exchanged every

two days. HUVECs were cultured with EGM-2 medium (Lonza), and hMSCs were maintained with mesenchymal stem cell growth medium (MSCGM, Lonza). To generate MGV-incorporated liver organoids, a 4 mg/mL collagen hydrogel containing MGVs was prepared using rat tail collagen type I (Corning). A pre-gel solution for the collagen hydrogel (final concentration: 4 mg/mL) was prepared by mixing the collagen solution with 10% (v/v) 10× PBS (Sigma-Aldrich) and 10% (v/v) MGVs ($OD_{500}$ = 16). Finally, the pH of the pre-gel solution was adjusted to 7.4 using 0.5 M sodium hydroxide (NaOH, Sigma-Aldrich). After centrifuging a Eppendorf tube containing the four types of cells at a ratio of 10:7:2:2 (hepatic endodermal cell:HUVEC:hMSC:hepatic stellate cell), the supernatant was removed and the pre-gel solution was added for cell resuspension. The final density of total cells was $2\times10^7$ cells/mL, and 5 μL of collagen hydrogel was used for the production of one liver organoid. The liver organoids generated in MGV-containing collagen hydrogels were cultured in ultra-low attachment 24-well plates (Corning) using the following medium: basal medium with equal volumes of HCM Bulletkit (Lonza) and EGM-2 medium supplemented with 3% (v/v) FBS, 1% (v/v) P/S, 20 ng/mL HGF (Peprotech), 10 ng/mL Oncostatin-M (ProSpec, Rehovot, Israel), and 0.1 μM dexamethasone.

**Immunostaining of organoids.** Whole-mount immunostaining was performed to check the overall protein expression in organoids. First, cultured organoids were fixed with 10% (v/v) formalin (Sigma-Aldrich) for 1 h at room temperature (RT), and then permeabilized with 0.1% (v/v) Triton X-100 (Sigma-Aldrich) for 30 minutes at RT. After blocking samples with 5% (w/v) bovine serum albumin (BSA, MP biomedical, Asse-Relegem, Belgium) solution for 2 h at RT, the organoid samples were incubated with primary antibodies for 24 h at 4 ℃. After washing three times with 1× PBS, secondary antibodies were added to the samples for 24 h at 4 ℃. After washing three times with 1× PBS, 4’,6-diamidino-2-phenylindole (DAPI, TCI Chemicals, Tokyo, Japan) was added to stain the nuclei of the organoids. Separately, immunostaining was performed after sectioning the organoids to more clearly identify the internal organoid structure. To this end, cultured organoids were fixed with 10% (v/v) formalin (Sigma-Aldrich) for 1 h at RT, and then optimal cutting temperature (OCT) blocks containing organoids were prepared using OCT compound (CellPath, Newtown, United Kingdom). After slicing the OCT blocks into sections, they were permeabilized with 0.1% (v/v) Triton X-100 for 10 minutes at RT. After blocking the samples with 5% (w/v) BSA solution for 2 h at RT, they were incubated with primary antibodies overnight at 4 ℃. After washing three times with 1× PBS, secondary antibodies were added to the samples for 1 h at RT. After washing three times with 1× PBS, DAPI was added to stain the nuclei of the organoid samples. In this study, the following primary antibodies were used: mouse monoclonal (B4) anti-smooth muscle actin (SMA, #sc-53142, 1:50, Santa Cruz Biotechnology, Inc., Dallas, TX, USA), mouse monoclonal (LN-6) anti-vimentin (VIM, #MAB1681, 1:50, Sigma-Aldrich), rabbit monoclonal (EPR5701) anti-P63 (#ab124762, 1:200, Abcam, Cambridge, MA, USA), mouse monoclonal (45M1) anti-MUC5AC (#ab3649, 1:200, Abcam), mouse monoclonal (6-11B-1) anti-acetylated α-tubulin (α-tubulin, #sc-23950, 1:50, Santa Cruz Biotechnology, Inc.), mouse monoclonal (C3) anti-AFP (#sc-8399, 1:50, Santa Cruz Biotechnology, Inc.), rabbit polyclonal anti-albumin (ALB, #A3293, 1:200, Sigma-Aldrich), rabbit monoclonal (28E1) anti-PDGF receptor β (PDGFRB, #3169, 1:100, Cell Signaling), mouse monoclonal (GA5) anti-glial fibrillary acidic protein (GFAP, #MAB3402, 1:200, Sigma-Aldrich), rabbit polyclonal anti-collagen type 1 (#234167, 1:50, Sigma-Aldrich) and mouse monoclonal (8B4) anti-MMP2 (#sc-13595, 1:50, Santa Cruz

Biotechnology, Inc). The following secondary antibodies were used; Alexa-Fluor 488-conjugated anti-mouse IgG (#A11001, 1:200, Thermo Fisher Scientific), Alexa-Fluor 488-conjugated anti-rabbit IgG (#A11008, 1:200, Thermo Fisher Scientific), Alexa-Fluor 594-conjugated anti-mouse IgG (#A11005, 1:200, Thermo Fisher Scientific), and Alexa-Fluor 594-conjugated anti-rabbit IgG (#A11012, 1:200, Thermo Fisher Scientific). TRITC-conjugated phalloidin (Sigma-Aldrich) for staining F-actin in the cytoskeleton was added to the samples treated with secondary antibodies. Stained organoids and sections were imaged using a confocal microscope (LSM 900, Carl Zeiss, Jena, Germany) and analyzed using Zen (Zeiss, version 3.0)

***In vitro* cytotoxicity assay**
HEK293T cells (American Type Culture Collection (ATCC), CLR-2316) were cultured in 24-well plates at 37 °C and 5% $CO_2$ in a humidified incubator in 0.5 ml of DMEM (Corning, 10-013-CV) with 10% FBS (Gibco) and 1× penicillin–streptomycin until about 80% confluency before experiment. For the cytotoxicity assay, HEK293T cells were cultured in DMEM with 10% FBS and penicillin/streptomycin and seeded in a 24-well plate. When the cells had reached 70-80% confluence, they were treated with MGVs and GVs in the concentrations desired. The plates were incubated in the incubator for 24 hours. Cytotoxicity was then measured using resazurin reduction (MTT) activity on cells treated with MGVs or GVs and compared to control cells, as directed by the manufacturers. A The Spark multimode microplate reader (Sparkcontrol, version 3.1 SP1, Tecan, Switzerland) was used to measure absorbance at 540 nm.

**Eukaryotic cell line authentication**
Lung organoids were authenticated with immunostaining of airway markers (P63, MUC5AC, α-tubulin). HEK293T-Rspo1-Fc cells were not authenticated after purchase. hiPSCs were authenticated with immunostaining of pluripotency markers (OCT4, TRA-1-60, SOX2) and alkaline phosphatase staining. HUVECs and hMSCs were authenticated by Lonza before delivery and not authenticated subsequently. HEK293T cells were authenticated by ATCC before delivery using short tandem repeat (STR) profiling.

**Preparation of superparamagnetic iron oxide nanoparticles conjugated GVs (SPIO-GVs)**
The superparamagnetic iron oxide nanoparticles were synthesized as previously described[6]. The SPIOs were coated and functionalized using the same strategy as MNPs. They were conjugated to GVs in the identical manner as MGVs. Inductively coupled plasma mass spectrometry (ICPMS, ICAP 7200 Duo + ASX-560, Thermo Fisher Scientific, USA) was used to determine the concentration of MNPs in SPIO-GVs.

**Acoustic signal attenuation measurement**
A fiber optic hydrophone was used to measure the acoustic response of our ultrasound signal, which was generated by an L22-14vX transducer with an 8 mm elevation focus and a center frequency of 15.625 MHz at desired voltage. We placed the fiber optic hydrophone (Precision Acoustics Ltd, version 1.2.0.27, Dorset, UK) beneath the transducer in the focus region. 5mm thick hydrogel phantoms (agarose, polyacrylamides), tissue embedded in 0.5% agarose were casted to 3D printed mold and were placed

between the hydrophone and ultrasound transducer. Reflection coefficients were calculated based on density and speed of sound [7–9].

**Acoustic scattering response measurement**

To determine the acoustic response of two constructs (GVs and MGVs), an L22-14vX transducer was used to transmit and receive the acoustic signals. The transmit signal was the same as in MMUS imaging with a central frequency of 15.625 MHz for 3 cycles at a voltage of 3 V. The transducer was immersed in a liquid sample of PBS containing the stated particles at a concentration of 0.1 nM. The received radio frequency (RF) signal was time-gated to extract only the scattering signals from the constructs. The Fast Fourier Transform (FFT) was performed on the time-gated received signal.

**Low-frequency ultrasound imaging**

For low-frequency ultrasound MMUS imaging, ultrasound images were acquired using a Verasonics Vantage programmable ultrasound scanning system with an L10-4v 128-element linear array transducer with a 0.3-mm pitch, an 18-mm elevation focus and a center frequency of 6.25 MHz with a voltage of 15V (Verasonics, Kirkland, WA). Imaging, data collection, image generation, and quantification were carried out in the same manner as described in the method section for MMUS imaging.

***In vivo* experiments**

**Immunoglobulins and blood chemistry assays.** All *in vivo* experiments were conducted in accordance with a protocol 1735 approved by the California Institute of Technology's Institutional Animal Care and Use Committee (IACUC). Animal housing room temperatures are monitored at all times and maintained between 71 and 75 degrees F for most species according to their physiological needs. Humidity is maintained between 30-70%. Light intensity and light cycle timing are carefully regulated and monitored in Caltech laboratory animal facilities. Automated light timers ensure a consistent light-dark cycle with 13 hours on and 11 hours off. Animals were randomly assigned to experimental groups by the animal facilities. Five 4-week-old male Balb/C mice received a tail vein injection of 2,280 pM of MGVs once per weak for 4 weeks. The concentration of GVs was matched to the concentration of MGVs. The control group received a same volume of saline injection. Every week, blood samples were collected from check bleeds for immunoglobulin assays. Immunoglobulins were tested according to the protocol provided by the manufacturer (IgG: 501240, 1:10, Cayman Chemical, MI, USA; IgM: 88-50470-22, 1:250, Thermo Fisher Scientific, OH, USA). On the final day, blood samples were collected from the abdominal aorta and centrifuged for serum separation. The alanine transaminase, aspartate transaminase, total bilirubin, total protein and albumin levels were analyzed using a biochemistry automatic analyzer (IDEXX, CA, USA).

**Histological analyses.** To determine the dispersion of MGVs, three 4-week-old male Balb/C mouse were intravenously injected with 2,280 pM of MGVs. After 5 minutes the mouse were euthanasized and liver was harvested for histological analysis. The organs were fixed in 10% neutral-buffered formalin and then submerged in 70% EtOH for storage. The fixed tissue was embedded in paraffin and sectioned. For iron staining analysis, the sections were immersed in Prussian Blue staining solution (abcam ab150674,

Cambridge, MA, USA) according to the protocol provided by the manufacturer. The images were examined under a laser scanning confocal microscope (Zeiss LSM880, Jena, Germany) and analyzed by Zen (Zeiss, version 3.0).

## Supplementary Notes 1: MGVs Measure Stiffness Independently from Other Factors

To examine whether MGVs could measure the stiffness of materials regardless of their pore sizes, MMUS imaging was carried out with polyacrylamide gels, which are widely used for stiffness tunable tissue phantoms with relatively similar pore sizes (70 nm ~ 130 nm). Additionally, MGVs with the size of (494.1 ± 11.5 nm) are unable to enter the pores of polyacrylamide gels, resulting in unaffected MMUS signals by the pore size. The stiffness of polyacrylamide gels could be varied from 74 Pa to 14 kPa. In addition, we created phantoms with similar densities by utilizing the same percentage of acrylamide (either 5% or 8%) while still achieving different levels of stiffness **(Supplementary Table 1)**. Consistent with the results from agarose gel phantoms, we observed an inverse relationship of MMUS signals as a function of increasing stiffness of the polyacrylamide phantoms while MGV concentrations and magnetic field strengths remain constant (**Extended Data Fig. 1a**). Furthermore, MGV-based MMUS imaging of two distinct hydrogels (agarose and polyacrylamide), which have the same elastic modulus but different compositions and pore sizes, generated highly similar MMUS signals, suggesting MMUS imaging could measure stiffness independent from material compositions and pore sizes **(Extended Data Fig. 1b)**.

## Supplementary Notes 2: Potential of MGV-Based MMUS Imaging for Clinical Translation

To expand MMUS imaging towards clinical translation, our MGV-based system needs to show compatibility with clinically relevant ultrasound frequencies and minimum *in vivo* toxicity. A lower-frequency ultrasound transducer (6.25 MHz) was able to detect the MMUS signals from MGVs, providing evidence that the MMUS system may be useful in diagnostic ultrasound **(Supplementary Fig. 12)**. Furthermore, to address the issues of biocompatibility posed by zinc-doped magnetic nanoparticles and enhance the clinical potential[6,10], we evaluated MMUS signal generated by GVs conjugated with conventional superparamagnetic iron oxide nanoparticles (SPIO-GVs). While MGVs produced stronger MMUS signals than SPIO-GVs due to higher magnetism of zinc-doped nanoparticles[6], SPIO-GVs still generated significantly higher MMUS signals than only GVs or only SPIOs **(Supplementary Fig. 13)**.

Finally, to make our system comparable across models, quantification methods were used to estimate the modulus of organoids and liver tissues. We established the correlation between the concentration of MGVs and the B-mode signal, as well as the relationship between the stiffness of the material and the MMUS signal. Using these correlations, we were able to estimate the concentration of MGVs in each sample and subsequently estimate their modulus by normalizing the MMUS signals to the MGV concentration of OD 1 **(Supplementary Fig. 14a)**. The measured MMUS signals to the elastic moduli and their elastic modulus of fibrotic organoids were about 2-fold larger (1.91- and 2.17-folds for MMUS imaging and rheometer, respectively) than that of normal organoids **(Supplementary Fig. 14a-b)**. The elastic modulus of the fixed liver samples measured by MMUS and rheometer were ~40- and ~84-fold greater than that of the normal liver samples **(Supplementary Fig. 14c-e)**. Our results establish the ability of our magneto-acoustic nanostructures to visualize and quantitatively detect tissue stiffness within the context of *in vivo* imaging.

## Supplementary Notes 3: Discussion

**Advantages of MGVs-based imaging in clinical applications**

As our results establish a new class of hybrid protein nanostructure (MGVs) as nanomaterial-based magneto-acoustically modulated MMUS contrast agents for non-invasive and sensitive imaging and measurement of tissue elasticity *in vivo*. The MGV-based imaging modality couples the improved ultrasound imaging sensitivity of gas vesicles with the capacity of MNPs enabling non-invasive, long-term, safe *in vivo* imaging to deepen our understanding of whole tissue mechanics, compared to prior MMUS imaging or fluorescence-based mechano-sensors[11,12]. The ability of our MGVs to visualize and measure stiffness in 3D tissues *in vivo* was demonstrated by successful detection and monitoring of fibrosis development and therapeutic effects in 3D organoid and *in vivo* models. Due to rapid uptake and accumulation of nanostructures by the liver, the clinical potential of MGVs for accurate and non-invasive *in vivo* detection of hepatic fibrosis could be readily tested for detecting hepatic fibrosis at an early stage and determining the efficacy of new anti-fibrosis therapies. A number of non-invasive techniques, such as magnetic resonance imaging (MRI) and shear-wave elastography, have been employed to detect fibrosis, such as fibrin-specific Gd-agents[10,13]. Due to ultrasound's wider availability and lower cost compared to MRI, MMUS imaging holds promise as a long-term disease monitoring technique. For example, after MRI-based early detection of fibrosis, therapeutic response to anti-fibrotic treatment can be monitored over time using regular MMUS imaging to evaluate long-term efficacy of the treatment.

While magnetic functionalization of microbubbles was developed to target and deliver these microbubbles to the desired location of vasculature, their micrometer size and short half-life limits their use for measuring internal tissue mechanical properties[14,15]. In comparison to intravascular microbubble-based ultrasound contrast agents, MGVs have distinct advantages in their superior functional and structural stability in physiological conditions and ability to penetrate extravascularly of certain tissues such as the liver. Given MGVs' increased tissue accessibility and ability to detect tissue stiffness *in vivo*, our MGV-based MMUS imaging can pave a way for a new strategy to ultrasound imaging-based diagnosis of a wide range of diseases.

**Potential improvements for more robust and broader measurement**

As the elastic modulus of tissues are a reliable biophysical parameter for disease diagnosis[16], further application-centric optimizations and development of larger magnetic field gradients, quantifiability, imaging acquisition and processing will help MGV-based imaging achieve widespread use. The MMUS system currently operates at a relatively low magnetic strength of 7-30 mT which is sufficient to cover a 1-cm field. However, to image deeper regions of the body in larger animals and humans, a stronger magnetic field over larger spatial scale may be necessary. Since our entire system costs less than $1,000, the simplest way to improve it would be to add a stronger magnet to the system at a low cost. In addition, we could consider combining our MGV-based MMUS imaging platform with clinically available strong magnetic fields, such as MRI or magnetic particle imaging, to create a diagnostic tool for clinical use with the benefits of ultrasound offering rapid, real-time, portable, and easy-to-use imaging. Moreover, quantifiability of MMUS imaging is important for making our system comparable across models. While we demonstrated the utility of MMUS imaging to determine the elastic moduli of organoids, more optimizations will be needed to improve the quantification of MGVs concentration in a broader range of

tissues *in vivo* because the AM signal from MGVs within living tissues may differ from MGVs of the identical concentration in *in vitro* phantom. With these improvements, we envision broad utility of our protein nanosturcture as a powerful mechanobiology research platform for quantifying tissue-scale mechanical properties and investigating the roles of these properties during the highly complex processes of development and disease progression.

**Unveiling tissue-level 3D mechanobiology insights using MGV-based imaging**

Recent advances in stem cell-derived organoid systems have provided a promising new class of biological models for understanding various human pathologies and screening of therapeutic drugs and targets[17]. While several recently developed materials could function as *in vivo* force sensors, long-term *in vivo* imaging of 3D mechanics still remains a challenge due to the large size of these materials, their limited stability and low detection sensitivity in deep tissue[12,18]. In this study, MGV-based imaging was successfully used to visualize mechanical dynamics of organoids by monitoring the progression of fibrosis and response to anti-fibrotic drugs for long-term. In particular, the ease of grafting MGVs into organoids by microinjection or encapsulation with the lack of toxicity and degradation, empowers a wide range of mechanobiology studies using organoid models of human diseases. Thus, it can be used to investigate the roles of tissue mechanics in diseases (e.g., metastatic cancer, developmental diseases) that involve changes in mechanical properties[19,20]. Aa a result, MGV-based measurement of mechanical dynamics may provide novel insights into the roles of mechanobiology in human disease that could be useful in tissue mechanics-based diagnosis and prediction of the best therapeutic outcomes.

## Supplementary Figures

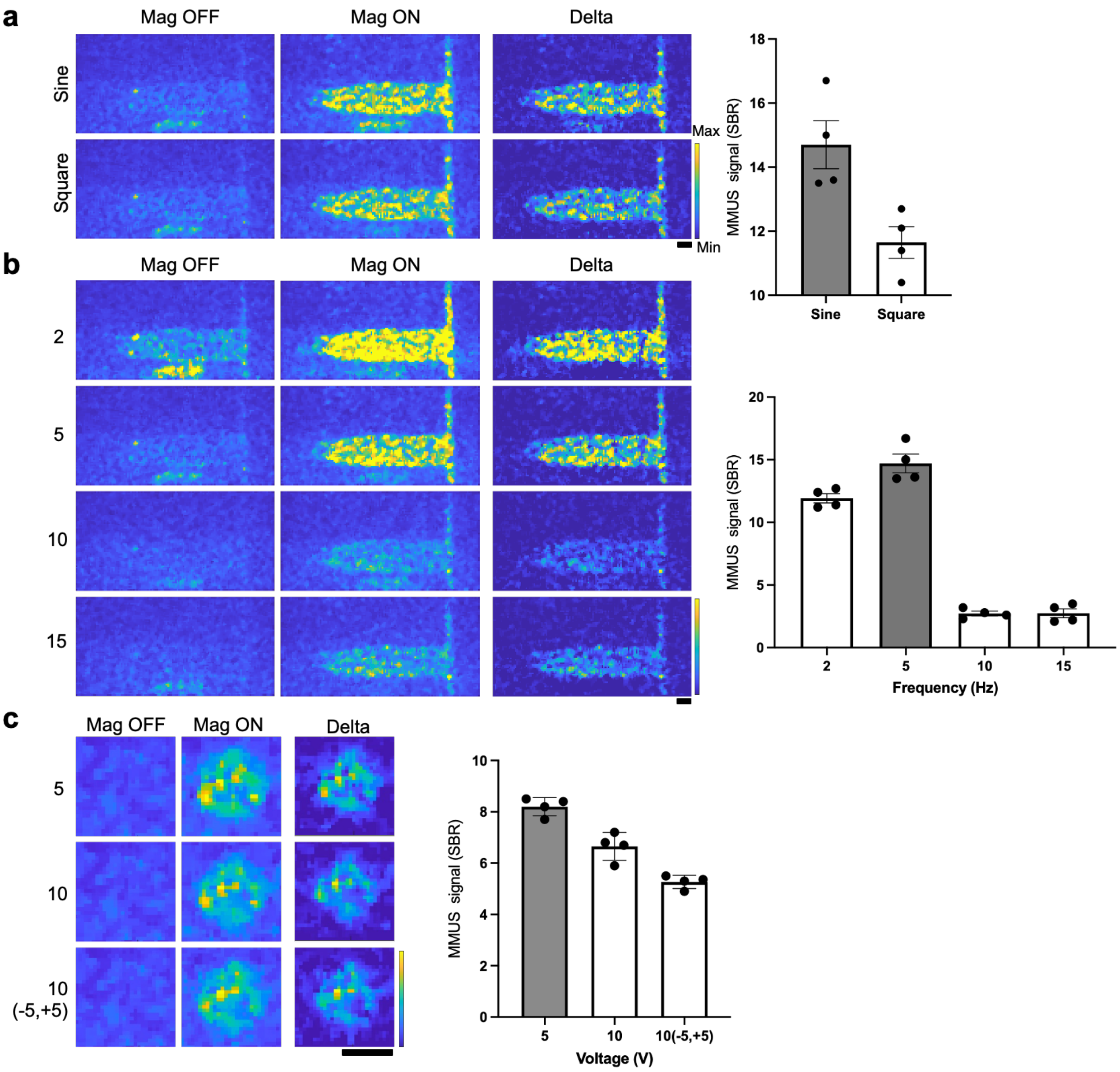


**Supplementary Figure 1. Optimization of magnetic properties using 5-micrometer magnetic particles.** (a) MMUS images and quantification of the sine and square wave forms (*n* = 4). (b) MMUS images and quantification using different magnetic frequencies (*n* = 4). (c) Images and quantification using different magnetic amplitudes (*n* = 4). All scale bars represent 1 mm. Min and max on the parula color bar represent 0 and 2000 arbitrary units, respectively. All points represent independent experiments, and line and error bars represent mean ± SEM.

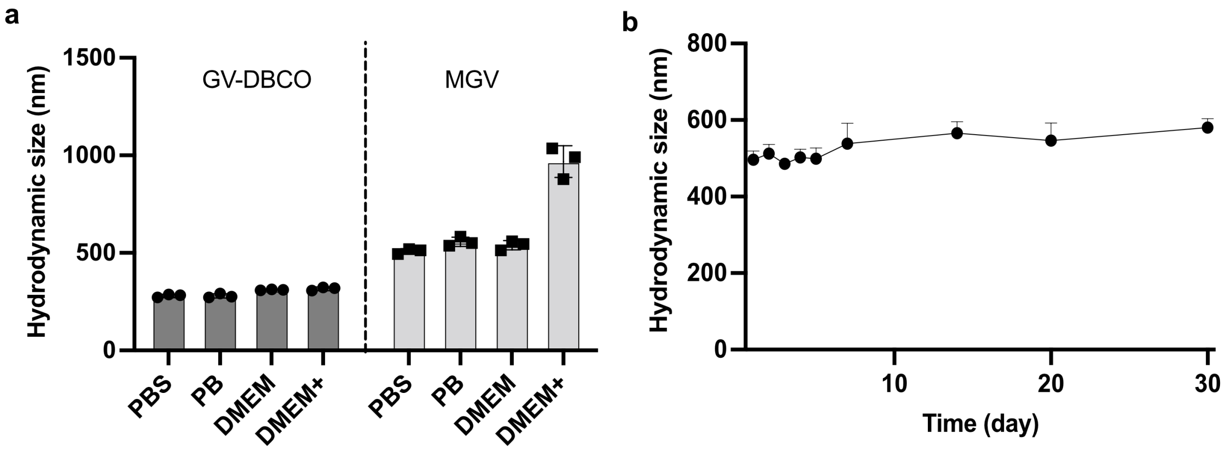


**Supplementary Figure 2. Stability of MGVs in various media.** Dynamic light scattering (DLS) analysis indicating the hydrodynamic size of MGVs (a) in different media conditions incubated for 1 h at 37°C (*n* = 3 independent experiments) and (b) in aqueous solution for 30 days at 4°C (*n* = 18 independent samples, number of trials = 3). Line and error bars represent mean ± SEM.

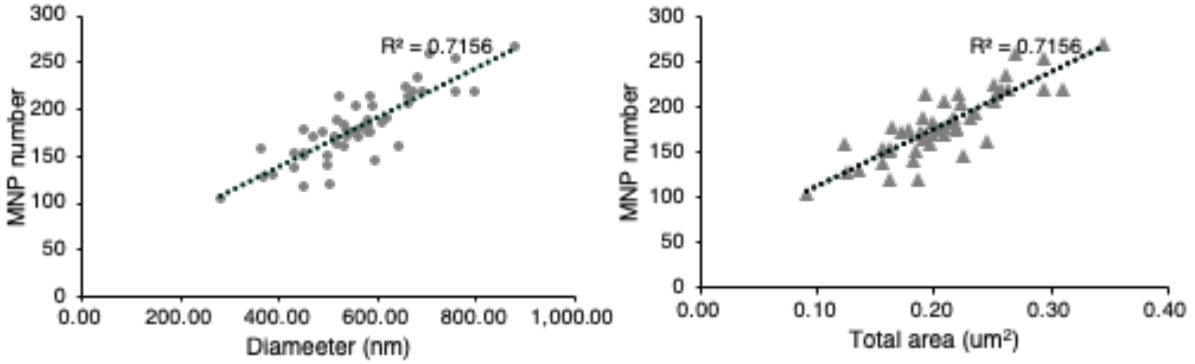


| | MNP Number | GV |
|---|---|---|
| Diameter | 186±34 | 581.3±124.7 nm |
| Area | 870 ± 116 | 1 μm³ |

**Supplementary Figure 3. Quantifying MNP numbers in MGVs.** Average MNP numbers in MGVs per GV diameter or area were calculated based on TEM images. MNPs were manually counted in individual TEM images of MGVs. ImageJ was used to calculate the GV diameter (*n* = 50 independent samples).

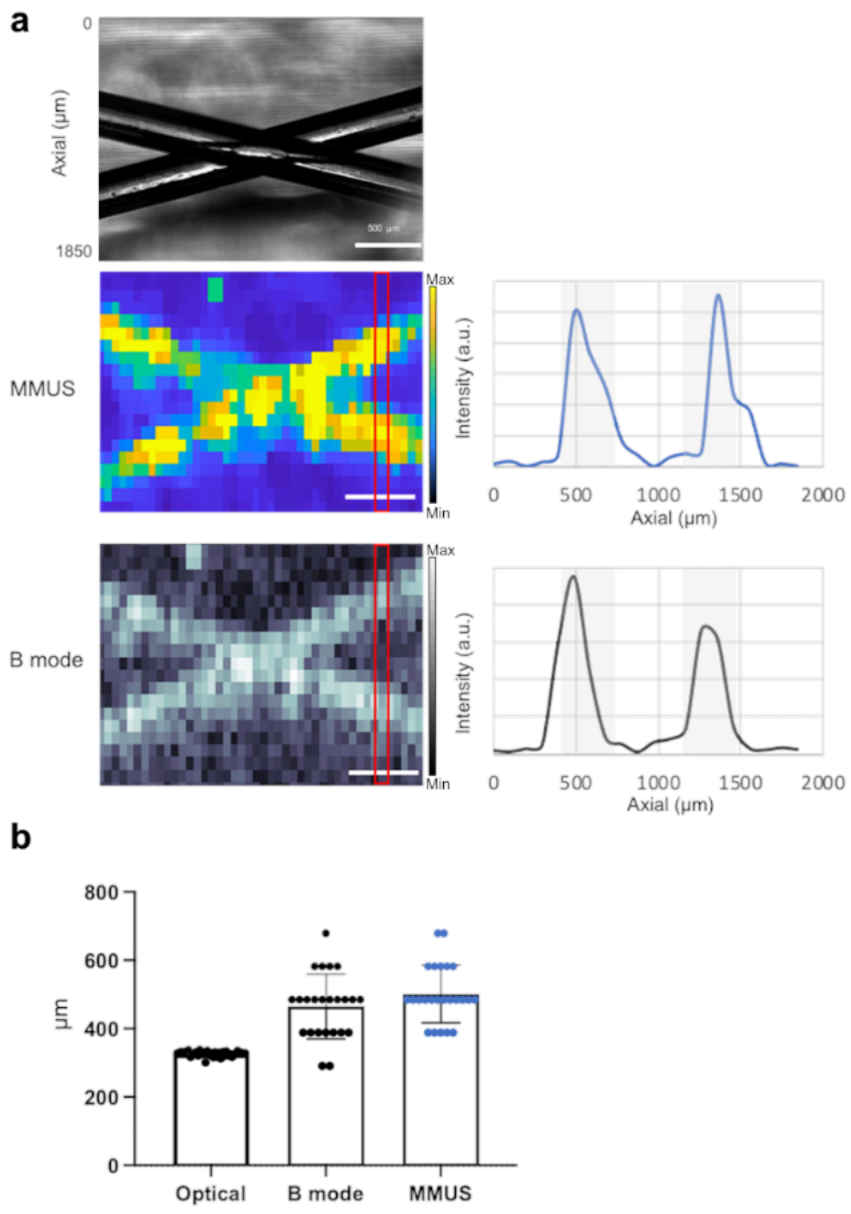


**Supplementary Figure 4. Spatial resolution estimation of MMUS and B mode imaging using an agar phantom with two cross lines of MGVs.** (a) Representative bright-field optical imaging of an agar phantom. The ground truth of the phantom thickness was quantified using ImageJ. Phantom MMUS and B mode images, and the intensity profiles, are shown for a vertical orientation. Scale bar: 500 µm. (b) Average line thickness of optical, B mode, and MMUS images. Min and max on color bars for parula and gray scale range from 0 to 10000 arbitrary units, respectively. Points represent 8 lines per images in *n* = 3 independent experiments, and line and error bars represent mean ± SD.

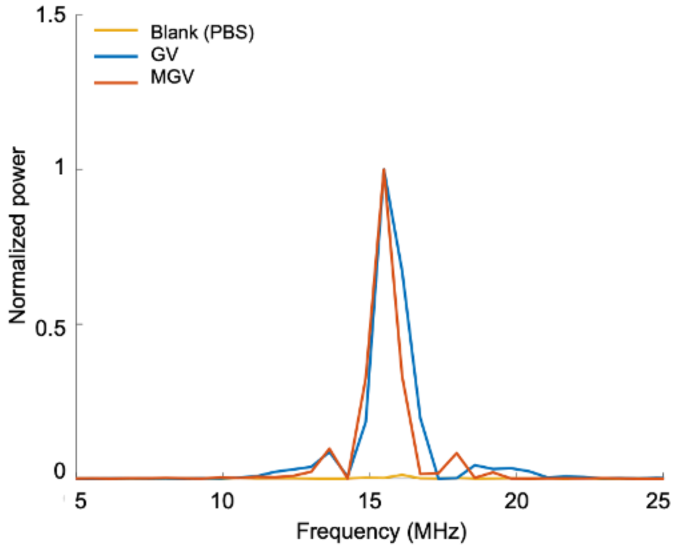


**Supplementary Figure 5. Acoustic frequency response of GVs and MGVs.** To measure the acoustic response the L22-14vX transducer was used to transmit and receive the signal. The transmit signal had a 15.625 MHz central frequency and a voltage of 3 V. Both GVs and MGVs were dispersed in 1X PBS solution at a concentration of 0.1 nM. GVs and MGVs showed similar acoustic responses with the central frequency at 15.6 MHz while the acoustic signals from PBS were negligible.

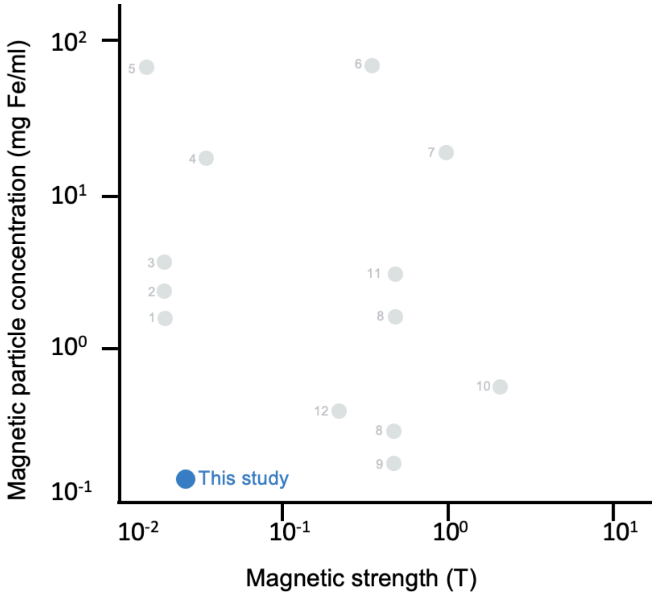


**Supplementary Figure 6. Comparison among previous MMUS studies.** The minimum concentration of magnetic particles (mg Fe/mL) and the minimum magnetic strength to achieve MMUS imaging in previous methods were compared to this study[21–32].

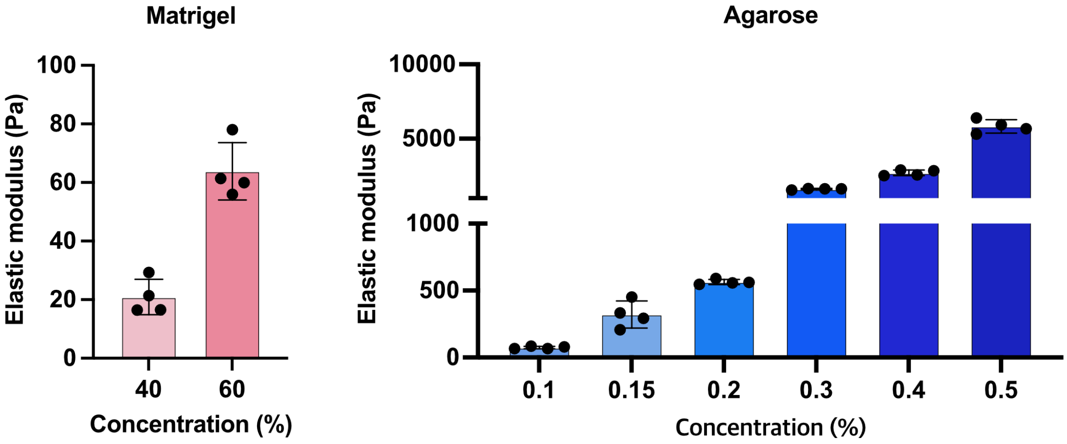


**Supplementary Figure 7. Elastic modulus of hydrogel materials.** Two types of hydrogel materials, Matrigel and agarose, were solidified using desired concentrations at 37 °C and 25 °C, respectively. The elastic modulus were determined using the rheometer storage modulus obtained at 1 Hz ($n$ = 4). All points represent independent experiments, and line and error bars represent mean ± SD.

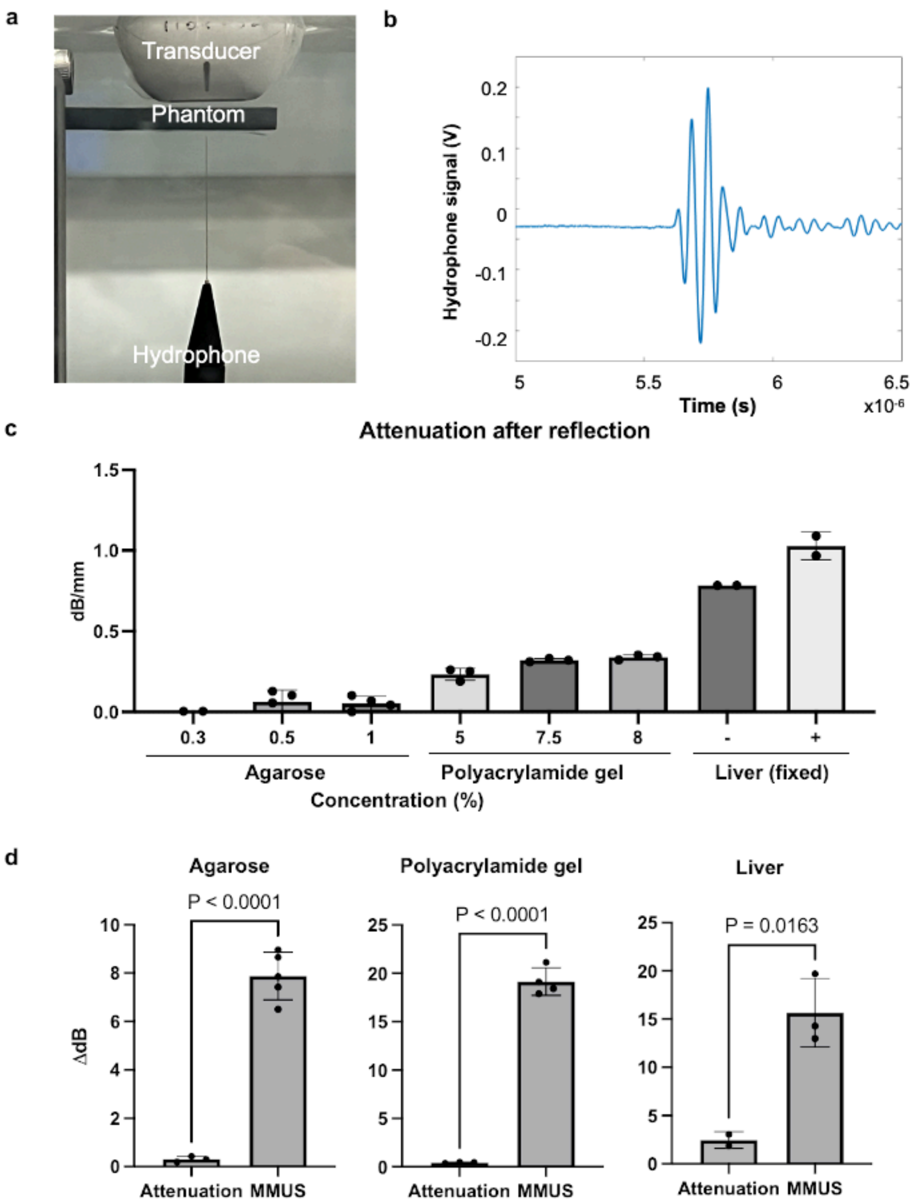


**Supplementary Figure 8. Ultrasound signal measurement using a hydrophone.** (a) Image of the experimental apparatus. The hydrophone was positioned 8 mm below the ultrasound transducer's focal region. (b) Representative trace of an ultrasound signal measured by the fiberoptic hydrophone at a frequency of 15.625 MHz. (c) Quantification of signal attenuation between various hydrogels and liver tissues. Independent samples are presented as dots (*n* = 3 for agarose and polyacrylamide gel, *n* = 2 for liver). (d) Quantification of the differences in signal attenuation and MMUS signals between the stiffest and softest variants of the same hydrogel or tissue material (*P* values left to right: 0.000013, 0.000003, 0.0163) (*n* = 3, 5 for agarose attenuation and MMUS, *n* = 3, 4 for polyacrylamide gel attenuation and MMUS, *n* = 2, 3 for liver attenuation and MMUS). All points represent independent experiments, and line and error bars represent mean ± SD. Significance was determined using the unpaired two-sided *t*-test.

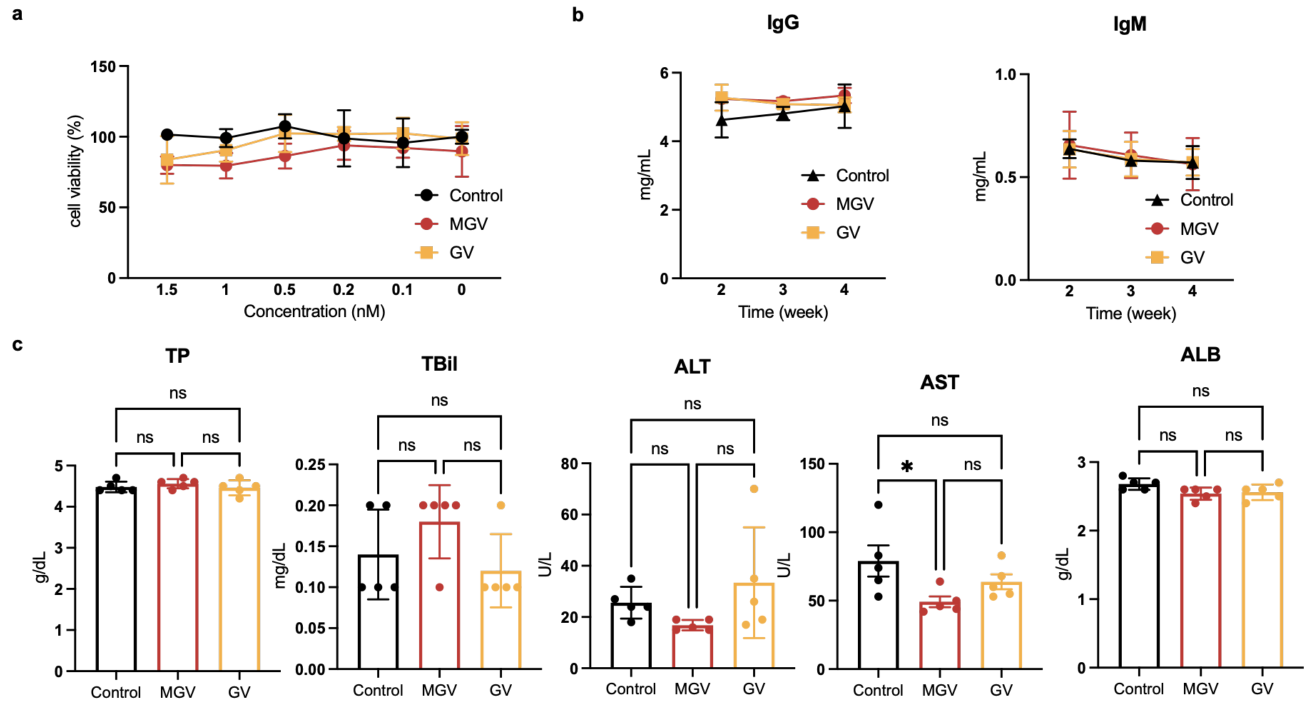


**Supplementary Figure 9. Immunogenicity and cytotoxicity test.** (a) *In vitro* cytotoxicity test. Relative viability of HEK293 cells treated with varying concentrations of GVs or MGVs (up to 1.5 nM) for 24 hours was tested ($n$ = 3 biological replicates). Line and error bars represent mean ± SEM, and significance was determined using one-way ANOVA with Tukey's multiple comparison test. (b) *In vivo* immunoglobulin level (IgG and IgM) ($n$ = 5 biologically independent animals for each group). Line and error bars represent mean ± SD, and significance was determined using two-way ANOVA with Tukey's multiple comparison test. (c) Blood chemistry assays. For liver function, total protein (TP), total-bilirubin (TBil), alanine transaminase (ALT), aspartate transaminase (AST), and albumin (ALB) were quantified. Line and error bars represent mean ± SD, and significance was determined using one-way ANOVA with Tukey's multiple comparison test. ($n$ = 5 biologically independent animals for each group); NS: not significant; *: $p<0.05$ ($P$ value: 0.0425).

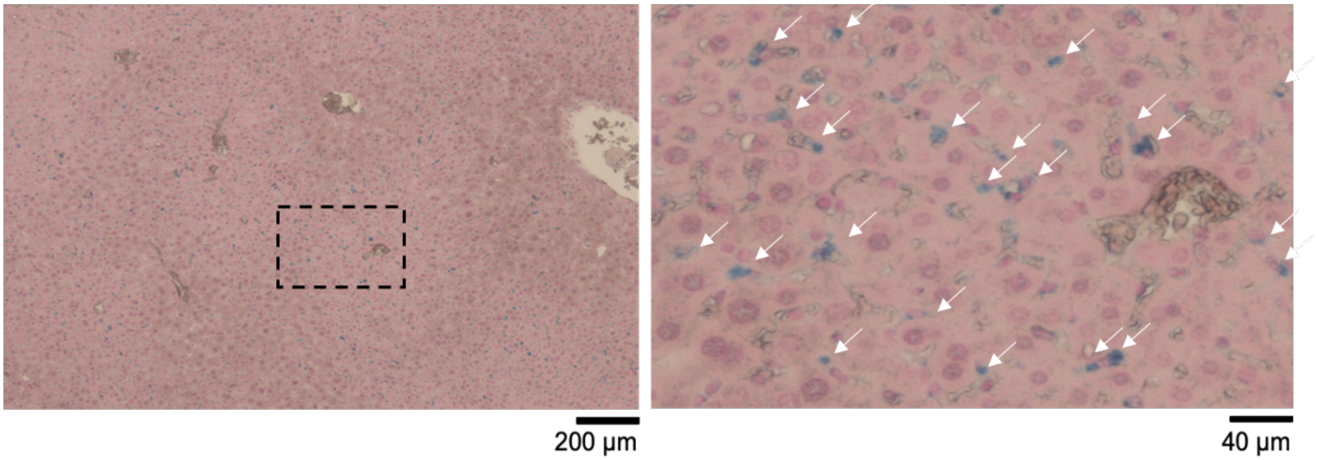


**Supplementary Figure 10. Particle dispersion following MGV intravenous injection into the tail vein.** Five minutes after injection, the liver was harvested and stained with Prussian blue (iron). ($n$ = 3 biologically independent animals). (scale bars = 200, 40 µm).

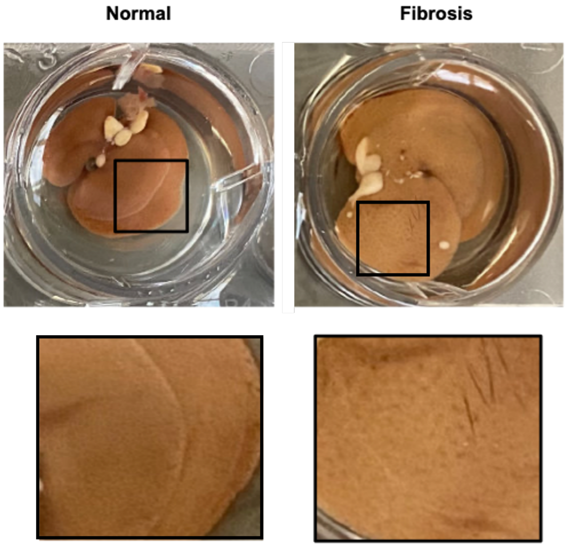


**Supplementary Figure 11. Differences in liver morphology between normal and fibrosis-induced liver organoid models.** Representative image of the liver for normal and fibrosis models treated with either mineral oil or $CCl_4$ (1 µL/g body weight), respectively. The liver surface was punctured and scarred on the outer surface of the liver organoid in the fibrosis group.

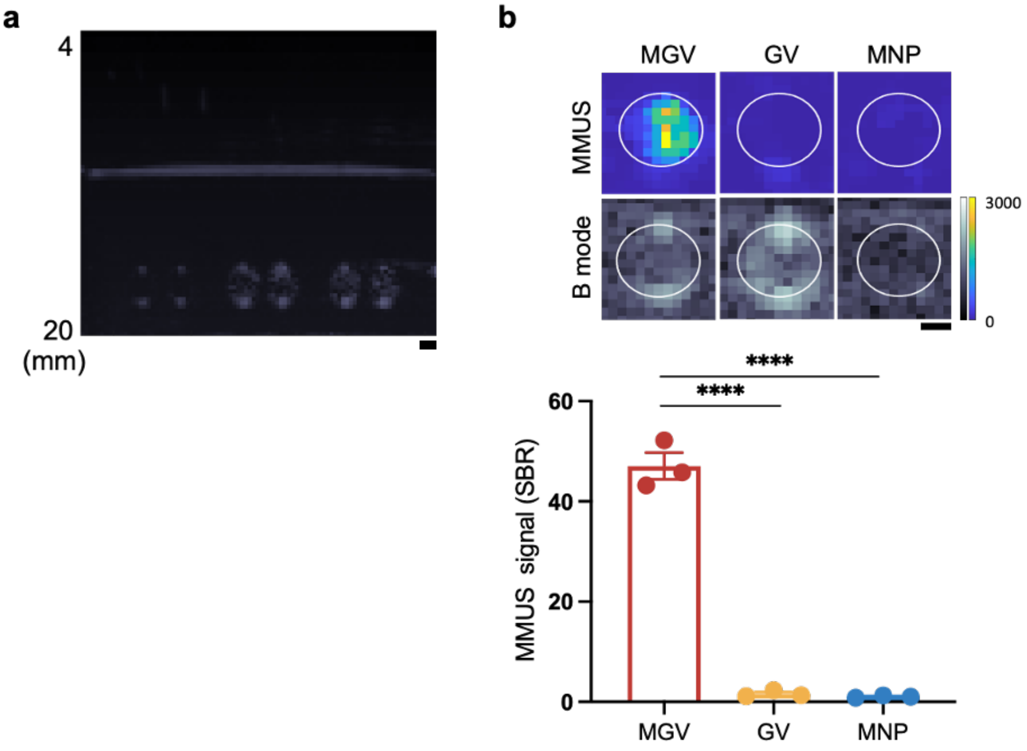


**Supplementary Figure 12. MMUS imaging with a low-frequency (6.25 MHz) ultrasound transducer (L10-4v).** (a) Representative B mode image of a lower frequency ultrasound transducer with an 18 mm focal region (*n* = 3 independent experiments). (b) MMUS images and SBR quantification of 0.8 nM MGVs, GVs, and MNPs in 0.1% (w/v) agarose phantom (*n* = 3 independent experiments). Scale bars represent 1 mm. Min and max on color bars for parula and gray scale range from 0 to 3000 arbitrary units, respectively. Line and error bars represent mean ± SEM, and significance was determined using one-way ANOVA with Tukey's multiple comparison test (*P* values left to right: 0.00000197, 0.00000186); ****: p<0.0001.

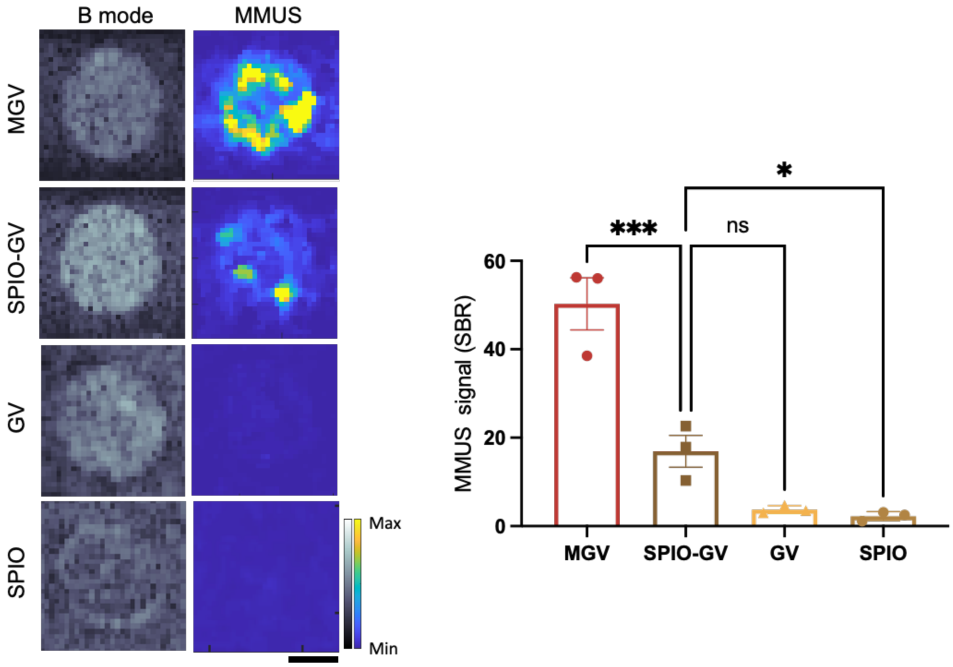


**Supplementary Figure 13. Comparison of MMUS imaging and SBR quantification between MGVs and superparamagnetic iron oxide nanoparticles conjugated GVs (SPIO-GV).** MGVs, SPIO-GVs, SPIO nanoparticles synthesized to contain the same amount of iron oxide (iron concentration 0.1 mg/ml) were used. The concentration of gas vesicles was matched to 0.2 nM ($n$ = 3 independent experiments). Scale bars represent 1 mm. Min and max on color bars for parula and gray scale range from 0 to 10000 arbitrary units, respectively. Line and error bars represent mean ± SEM, and significance was determined using one-way ANOVA with Dunnett's multiple comparison test ($P$ values left to right: 0.000388, 0.066004, 0.041961); *: $p<0.05$; **: $p<0.01$; ***: $p<0.001$.

a

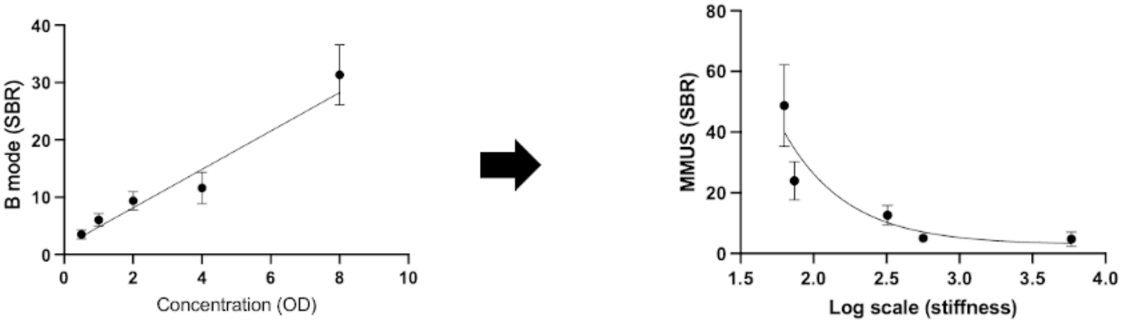


e.g.

| B mode signal | OD |
|---|---|
| 58.97 | 16 |

| MMUS signal per OD 1 | Stiffness (log scale) | Stiffness (Pa) |
|---|---|---|
| 25.5 | 1.98 | 95.5 |

b

(pa)

| Sample | | Normal | Drug | Fibrosis |
|---|---|---|---|---|
| Organoid | Lung | 250.1 | 549.5 | 2951.2 |
| | Liver | 95.5 | 162.1 | 181.9 |

c

(pa)

| | Normal | Fibrosis |
|---|---|---|
| MMUS | 95.5 ± 13.3 | 181.9 ± 40.6 |
| Rheometer | 167.9 ± 33.7 | 365.6 ± 77.7 |

d

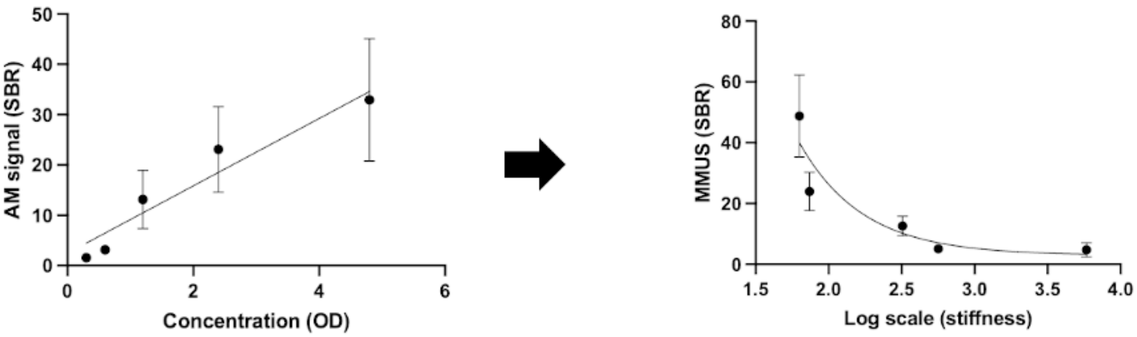


e

Mouse liver

| | Normal | Fixed |
|---|---|---|
| MMUS | 574.4 ± 62.9 | 21801.6 ± 15881.8 |
| Rheometer | 917.9.1 ± 124.5 | 77282.4 ± 19360.4 |

**Supplementary Figure 14. Estimating elastic modulus from MMUS images.** (a) (left) A linear standard curve for the relationship between MGV concentrations and B mode signal (SBR) ($n$ = 3 independent experiments). (right) A non-linear standard curve for the relationship between stiffness and MMUS signal ($n$ = 5 independent experiments). The modulus of various biological systems can be calculated based on the above two curves. For example, if one sample has a B mode signal of 58.97 and an MMUS signal of

408, we can estimate MGVs concentration to be OD 16 using the standard curve. The sample's stiffness can be calculated by dividing the MMUS signal down to OD 1. (b) Estimation of the stiffness of liver and lung organoids based on MGV-based MMUS imaging. (c) A comparison of the stiffness of liver organoids in normal and fibrosis obtained using MMUS imaging and rheometer ($N$ = 3 for MMUS, $N$ = 5 for rheometer independent samples). (d) xAM signal (SBR) dependent to various MGV concentrations and MMUS signal (SBR) dependent with various stiffness used in (a) (left: $n$ = 3, right: $n$ = 5 independent experiments). (e) Comparison of the stiffness obtained using MMUS and rheometer of normal and fixed liver ($N$ = 3 for MMUS, $N$ = 5 for rheometer independent samples). For (a, d) continuous lines are best fitting curves to the data with a linear or exponential model, dots and error bars represent mean ± SD.

## Supplementary Table

**Supplementary Table 1. Phantom formulations utilizing stiffness-varying polyacrylamides**

| Acrylamide % | Bis-acrylamide % | 40% Acrylamide (ml) | 2% Bis-acrylamide (ml) | Water* (ml) | E ± SD (Pa) |
|---|---|---|---|---|---|
| 5 | 0.06 | 1.25 | 0.30 | 8.45 | 74.0 ± 23.4 |
| 5 | 0.3 | 1.25 | 1.50 | 7.25 | 2511.7 ± 330.2 |
| 7.5 | 0.24 | 1.875 | 1.18 | 6.945 | 5299.2 ± 515.0 |
| 8 | 0.048 | 2.00 | 0.24 | 7.76 | 637.9 ± 237.3 |
| 8 | 0.48 | 2.00 | 2.40 | 5.60 | 14095.8 ± 2947.2 |

*0.002 M lithium phenyl-2,4,6-trimethylbenzoylphosphinate (LAP) solution dissolved in DIW

# Supplementary References


1. Sachs, N. *et al.* Long-term expanding human airway organoids for disease modeling. *EMBO J* **38**, e100300 (2019).
2. C, L., W, X., C, G. & Y, D. Pronuclear microinjection and oviduct transfer procedures for transgenic mouse production. *Methods Mol Biol* **1027**, 217–232 (2013).
3. T, T. *et al.* Massive and Reproducible Production of Liver Buds Entirely from Human Pluripotent Stem Cells. *Cell Rep* **21**, 2661–2670 (2017).
4. Takebe, T. *et al.* Vascularized and functional human liver from an iPSC-derived organ bud transplant. *Nature 2013 499:7459* **499**, 481–484 (2013).
5. M, C. *et al.* Generation of Hepatic Stellate Cells from Human Pluripotent Stem Cells Enables In Vitro Modeling of Liver Fibrosis. *Cell Stem Cell* **23**, 101-113.e7 (2018).
6. Jang, J. T. *et al.* Critical enhancements of MRI contrast and hyperthermic effects by dopant-controlled magnetic nanoparticles. *Angew Chem Int Ed Engl* **48**, 1234–1238 (2009).
7. Ben Cox. Acoustics for Ultrasound Imaging Contents. (2013).
8. Chen, P. *et al.* Acoustic characterization of tissue-mimicking materials for ultrasound perfusion imaging research. *Ultrasound Med Biol* **48**, 124–142 (2022).
9. Zell, K., Sperl, J. I., Vogel, M. W., Niessner, R. & Haisch, C. Acoustical properties of selected tissue phantom materials for ultrasound imaging. *Phys Med Biol* **52**, (2007).
10. Shuvaev, S., Akam, E. & Caravan, P. Molecular MR Contrast Agents. *Invest Radiol* **56**, 20–34 (2021).
11. Sjöstrand, S., Evertsson, M. & Jansson, T. Magnetomotive Ultrasound Imaging Systems: Basic Principles and First Applications. *Ultrasound in Medicine and Biology* vol. 46 2636–2650 Preprint at https://doi.org/10.1016/j.ultrasmedbio.2020.06.014 (2020).
12. Lee, W. *et al.* Dispersible hydrogel force sensors reveal patterns of solid mechanical stress in multicellular spheroid cultures. *Nature Communications 2019 10:1* **10**, 1–14 (2019).
13. Vorobiev, V. *et al.* Vascular-targeted micelles as a specific MRI contrast agent for molecular imaging of fibrin clots and cancer cells. *Eur J Pharm Biopharm* **158**, 347–358 (2021).
14. Beguin, E., Bau, L., Shrivastava, S. & Stride, E. Comparing Strategies for Magnetic Functionalization of Microbubbles. *ACS Appl Mater Interfaces* **11**, 1829–1840 (2019).
15. Owen, J. *et al.* Magnetic targeting of microbubbles against physiologically relevant flow conditions. *Interface Focus* **5**, 1–12 (2015).
16. Wells, R. G. Tissue mechanics and fibrosis. *Biochimica et Biophysica Acta - Molecular Basis of Disease* vol. 1832 884–890 Preprint at https://doi.org/10.1016/j.bbadis.2013.02.007 (2013).
17. Kim, J., Koo, B.-K. & Knoblich, J. A. Human organoids: model systems for human biology and medicine. *Nature Reviews Molecular Cell Biology 2020 21:10* **21**, 571–584 (2020).
18. Mok, S. *et al.* Mapping cellular-scale internal mechanics in 3D tissues with thermally responsive hydrogel probes. *Nat Commun* **11**, (2020).
19. Martinez-Vidal, L. *et al.* Causal contributors to tissue stiffness and clinical relevance in urology. *Communications Biology 2021 4:1* **4**, 1–16 (2021).
20. Lee, G., Han, S. B., Lee, J. H., Kim, H. W. & Kim, D. H. Cancer Mechanobiology: Microenvironmental Sensing and Metastasis. *ACS Biomater Sci Eng* **5**, 3735–3752 (2019).
21. Pope, A. G. *et al.* Contrast-enhanced imaging of SPIO-labeled platelets using magnetomotive ultrasound. *Phys Med Biol* **58**, 7277–7290 (2013).
22. Levy, B. E. *et al.* Effect of Model Thrombus Volume and Elastic Modulus on Magnetomotive Ultrasound Signal Under Pulsatile Flow. *IEEE Trans Ultrason Ferroelectr Freq Control* **65**, 1380–1388 (2018).
23. Hossain, M. M., Levy, B. E., Thapa, D., Oldenburg, A. L. & Gallippi, C. M. Blind Source Separation-Based Motion Detector for Imaging Super-Paramagnetic Iron Oxide (SPIO) Particles in Magnetomotive Ultrasound Imaging. *IEEE Trans Med Imaging* **37**, 2356–2366 (2018).
24. Huang, P. C. *et al.* Magnetomotive Optical Coherence Elastography for Magnetic Hyperthermia Dosimetry Based on Dynamic Tissue Biomechanics. *IEEE J Sel Top Quantum Electron* **22**, 104–119 (2016).
25. Ahmad, A. *et al.* Magnetomotive optical coherence elastography using magnetic particles to induce mechanical waves. *Biomedical Optics Express, Vol. 5, Issue 7, pp. 2349-2361* **5**, 2349–2361 (2014).
26. Shen, W. H. & Li, M. L. Toward Real Time Backward-Mode Pulsed Magnetomotive Ultrasound. *IEEE International Ultrasonics Symposium, IUS* **2019-October**, 396–398 (2019).
27. Mazon, E. E. *et al.* A pulsed magnetomotive ultrasound imaging system for magnetic nanoparticle detection. *LAUS 2021 - 2021 IEEE UFFC Latin America Ultrasonics Symposium, Proceedings* (2021) doi:10.1109/LAUS53676.2021.9639204.

28. Qu, M., Mehrmohammadi, M. & Emelianov, S. Y. Sensing the delivery and endocytosis of nanoparticles using magneto-photo-acoustic imaging. *Photoacoustics* **3**, 107–113 (2015).
29. Mehrmohammadi, M. *et al.* Pulsed magneto-motive ultrasound imaging to detect intracellular accumulation of magnetic nanoparticles. *Nanotechnology* **22**, 415105 (2011).
30. Sjostrand, S. *et al.* Contrast-enhanced magnetomotive ultrasound imaging (CE-MMUS) for colorectal cancer staging: Assessment of sensitivity and resolution to detect alterations in tissue stiffness. *IEEE International Ultrasonics Symposium, IUS* **2019-October**, 1077–1080 (2019).
31. Oldenburg, A. L. & Boppart, S. A. Resonant acoustic spectroscopy of soft tissues using embedded magnetomotive nanotransducers and optical coherence tomography. *Phys Med Biol* **55**, 1189–1201 (2010).
32. Mehrmohammadi, M., Oh, J., Mallidi, S. & Emelianov, S. Y. Pulsed Magneto-motive Ultrasound Imaging Using Ultrasmall Magnetic Nanoprobes. *Mol Imaging* **10**, 102 (2011).
