# Supplementary material for: Magneto-acoustic protein nanostructures for non-invasive imaging of tissue mechanics in vivo
Source: Nat Mater. 2023 Oct 16;23(2):290–300. doi: 10.1038/s41563-023-01688-w (PMC10837075; doi:10.1038/s41563-023-01688-w)
Supplement: Supplementary file 2 — Reporting Summary [file 41563_2023_1688_MOESM2_ESM.pdf]

nature portfolio

Corresponding author(s): Jinwoo Cheon

Last updated by author(s): Jul 23, 2023

Reporting Summary

Nature Portfolio wishes to improve the reproducibility of the work that we publish. This form provides structure for consistency and transparency in reporting. For further information on Nature Portfolio policies, see our Editorial Policies and the Editorial Policy Checklist.

Statistics

For all statistical analyses, confirm that the following items are present in the figure legend, table legend, main text, or Methods section.

n/a	Confirmed	
☐	☒	The exact sample size (*n*) for each experimental group/condition, given as a discrete number and unit of measurement
☐	☒	A statement on whether measurements were taken from distinct samples or whether the same sample was measured repeatedly
☐	☒	The statistical test(s) used AND whether they are one- or two-sided<br>*Only common tests should be described solely by name; describe more complex techniques in the Methods section.*
☐	☒	A description of all covariates tested
☐	☒	A description of any assumptions or corrections, such as tests of normality and adjustment for multiple comparisons
☐	☒	A full description of the statistical parameters including central tendency (e.g. means) or other basic estimates (e.g. regression coefficient) AND variation (e.g. standard deviation) or associated estimates of uncertainty (e.g. confidence intervals)
☐	☒	For null hypothesis testing, the test statistic (e.g. *F*, *t*, *r*) with confidence intervals, effect sizes, degrees of freedom and *P* value noted<br>*Give P values as exact values whenever suitable.*
☒	☐	For Bayesian analysis, information on the choice of priors and Markov chain Monte Carlo settings
☒	☐	For hierarchical and complex designs, identification of the appropriate level for tests and full reporting of outcomes
☒	☐	Estimates of effect sizes (e.g. Cohen's *d*, Pearson's *r*), indicating how they were calculated

Our web collection on statistics for biologists contains articles on many of the points above.

Software and code

Policy information about availability of computer code

Data collection	We used MATLAB (version 2017b or 2020a, Mathworks) custom scripts, with functions provided by the Vantage 4.0.0 or 4.2.0 system (Verasonics), to acquire ultrasound images. All custom code will be available on the Shapiro Lab GitHub (http://github.com/shapiro-lab) upon publication. Software for microscopy were Ocular (version 2.0,Olympus Life Science), EVOS® FL Auto Cell Imaging System (software v.16) and Zen (version 3.0, Zeiss). Software for material characterizations were collected by commercial softwares (e.g., TEM, DigitalMicrograph 3.22.1461.0; VSM, Lake Shore VSM software 4.9.0; ICP-OES, Qtegra 2.6.2270.44; DLS, Zetasizer Software 7.12). For whole liver morphology iphone 11pro was used. Rheometer software RheoCompassTM (version V1.24.549, Anton Paar) was used. Software for microplate reader is Sparkcontrol (version 3.1 SP1, Tecan). Fiber optic hydrophone version is 1.2.0.27(Precision Acoustics Ltd).
Data analysis	We used MATLAB (2019a or 2022a, Mathworks) and Prism (version 9, Graphpad) for data and image analysis and plotting. For microscopic imaging analysis Zen (version 3.0, Zeiss), ImageJ (version 1.51j8, NIH) and OlyVIA (version 2.9, Olympus Life Science) was used. For material characterization analysis commercial softwares were used (e.g., TEM, DigitalMicrography 3.22.1461.0; VSM, Lake Shore VSM Software 4.9.0). Illustrations were made in Affinity Designer (version 1.10.0, Serif Europe).

For manuscripts utilizing custom algorithms or software that are central to the research but not yet described in published literature, software must be made available to editors and reviewers. We strongly encourage code deposition in a community repository (e.g. GitHub). See the Nature Portfolio guidelines for submitting code & software for further information.

Data

Policy information about availability of data

All manuscripts must include a data availability statement. This statement should provide the following information, where applicable:

- Accession codes, unique identifiers, or web links for publicly available datasets
- A description of any restrictions on data availability
- For clinical datasets or third party data, please ensure that the statement adheres to our policy

The data presented in this study are available in the Source data. Additional information and requests for resources and reagents that support the findings of this study are available from the corresponding author upon reasonable request. Source data are provided with this paper.

Human research participants

Policy information about studies involving human research participants and Sex and Gender in Research.

Reporting on sex and gender	To produce lung organoids, fragmented lung tissues were obtained from one male and two female patients. This study was not related to sex and gender and sex-specific analyses were not performed.
Population characteristics	Fragmented human lung tissues from anonymous patients who aged 19 to 80 (male or female) were obtained.
Recruitment	Tissue fragments were collected from patients undergoing lung surgery after acquiring their informed consents. There was no pre-selection on the patients and tissue fragments for lung organoid generation.
Ethics oversight	The use of human lung tissues for lung organoid generation was approved by the Institutional Review Board (IRB) of Severance Hospital (IRB No: 4-2021-1555).

Note that full information on the approval of the study protocol must also be provided in the manuscript.

Field-specific reporting

Please select the one below that is the best fit for your research. If you are not sure, read the appropriate sections before making your selection.

☒ Life sciences ☐ Behavioural & social sciences ☐ Ecological, evolutionary & environmental sciences

For a reference copy of the document with all sections, see nature.com/documents/nr-reporting-summary-flat.pdf

Life sciences study design

All studies must disclose on these points even when the disclosure is negative.

Sample size	The numbers of biologival and technical replicates were chosen based on preliminary experiments, so as to provide sufficient power for statistical comparison.
Data exclusions	No data were excluded from this study.
Replication	Replicates are reported in the figure legends.
Randomization	Standardized cell culture conditions and samples used in each set of experiments were equal to minimize variation across samples, except the experimental condition being tested. Cultured lung and liver organoids were randomly assigned for each group when they reached the each specified time point. Animals were randomly distributed into cages and ear-punched by animal care staff. Cages of animals were randomly chosen for the experimental groups versus control conditions. In all other experiments, samples were allocated randomly and performed with appropriate control.
Blinding	Blinding was not applicable to our study because our experiments did not involve human participants and was not possible since the main researcher was responsible for both data acquisition and analysis. All data collection, processing, and analysis methods were quantitative and identical across experimental groups.

Reporting for specific materials, systems and methods

We require information from authors about some types of materials, experimental systems and methods used in many studies. Here, indicate whether each material, system or method listed is relevant to your study. If you are not sure if a list item applies to your research, read the appropriate section before selecting a response.

Materials & experimental systems

n/a	Involved in the study	
☐	☒	Antibodies
☐	☒	Eukaryotic cell lines
☒	☐	Palaeontology and archaeology
☐	☒	Animals and other organisms
☒	☐	Clinical data
☒	☐	Dual use research of concern

Methods

n/a	Involved in the study	
☒	☐	ChIP-seq
☒	☐	Flow cytometry
☒	☐	MRI-based neuroimaging

Antibodies

Antibodies used

The following primary antibodies were used:
Mouse monoclonal (B4) anti-smooth muscle actin (SMA, #sc-53142, 1:50, Santa Cruz Biotechnology)
Mouse monoclonal (LN-6) anti-vimentin (VIM, #MAB1681, 1:50, Sigma-Aldrich)
Rabbit monoclonal (EPR5701) anti-P63 (#ab124762, 1:200, Abcam)
Mouse monoclonal (45M1) anti-MUC5AC (#ab3649, 1:200, Abcam)
Mouse monoclonal (6-11B-1) anti-acetylated α-tubulin (#sc-23950, 1:50, Santa Cruz Biotechnology)
Mouse monoclonal (C3) anti-AFP (#sc-8399, 1:50, Santa Cruz Biotechnology)
Rabbit polyclonal anti-albumin (ALB, #A3293, 1:200, Sigma-Aldrich)
Rabbit monoclonal (28E1) anti-PDGF receptor β (PDGFRB, #3169, 1:100, Cell Signaling)
Mouse monoclonal (GA5) anti-glial fibrillary acidic protein (GFAP, #MAB3402, 1:200, Sigma-Aldrich)
Rabbit polyclonal anti-collagen type 1 (#234167, 1:50, Sigma-Aldrich)
Mouse monoclonal (8B4) anti-MMP2 (#sc-13595, 1:50, Santa Cruz Biotechnology)
Goat Anti-Mouse IgG monoclonal, Mouse anti-IgG/HRP conjugate polyclonal(#501240, Cayman chemical)
Mouse anti-IgM monoclonal, HRP-conjugated anti-mouse Ig(H+L) polyclonal (#88-50470-22, Thermo Fisher Scientific)

The following secondary antibodies were used:
Alexa-Fluor 488-conjugated anti-mouse IgG (#A11001, 1:200, Thermo Fisher Scientific)
Alexa-Fluor 488-conjugated anti-rabbit IgG (#A11008, 1:200, Thermo Fisher Scientific)
Alexa-Fluor 594-conjugated anti-mouse IgG (#A11005, 1:200, Thermo Fisher Scientific)
Alexa-Fluor 594-conjugated anti-rabbit IgG (#A11012, 1:200, Thermo Fisher Scientific)

Validation

All antibodies listed above are commercially available and have been verified by many references provided on the website of the companies that sell antibodies (links below).

Mouse monoclonal (B4) anti-smooth muscle actin (SMA, #sc-53142, 1:50, Santa Cruz Biotechnology)
Validation Refs. from the manufacturer's datasheet: https://datasheets.scbt.com/sc-53142.pdf

Mouse monoclonal (LN-6) anti-vimentin (VIM, #MAB1681, 1:50, Sigma-Aldrich)
Validation Refs. from the manufacturer's datasheet: https://www.sigmaaldrich.com/product/mm/mab1681

Rabbit monoclonal (EPR5701) anti-P63 (#ab124762, 1:200, Abcam)
Validation Refs. from the manufacturer's datasheet: https://www.abcam.com/products/primary-antibodies/p63-antibody-epr5701-ab124762.html

Mouse monoclonal (45M1) anti-MUC5AC (#ab3649, 1:200, Abcam)
Validation Refs. from the manufacturer's datasheet: https://www.abcam.com/products/primary-antibodies/mucin-5ac-antibody-45m1-ab3649.html

Mouse monoclonal (6-11B-1) anti-acetylated α-tubulin (#sc-23950, 1:50, Santa Cruz Biotechnology)
Validation Refs. from the manufacturer's datasheet: https://datasheets.scbt.com/sc-23950.pdf

Mouse monoclonal (C3) anti-AFP (#sc-8399, 1:50, Santa Cruz Biotechnology)
Validation Refs. from the manufacturer's datasheet: https://datasheets.scbt.com/sc-8399.pdf

Rabbit polyclonal anti-albumin (ALB, #A3293, 1:200, Sigma-Aldrich)
Validation Refs. from the manufacturer's datasheet: https://www.sigmaaldrich.com/product/sigma/a3293

Rabbit monoclonal (28E1) anti-PDGF receptor β (PDGFRB, #3169, 1:100, Cell Signaling)
Validation Refs. from the manufacturer's datasheet: https://www.cellsignal.com/products/primary-antibodies/pdgf-receptor-b-28e1-rabbit-mab/3169

Mouse monoclonal (GA5) anti-glial fibrillary acidic protein (GFAP, #MAB3402, 1:200, Sigma-Aldrich)
Validation Refs. from the manufacturer's datasheet: https://www.sigmaaldrich.com/product/mm/mab3402

Rabbit polyclonal anti-collagen type 1 (#234167, 1:50, Sigma-Aldrich)
Validation Refs. from the manufacturer's datasheet: https://www.sigmaaldrich.com/product/mm/234167

Mouse monoclonal (8B4) anti-MMP2 (#sc-13595, 1:50, Santa Cruz Biotechnology)
Validation Refs. from the manufacturer's datasheet: https://datasheets.scbt.com/sc-13595.pdf

Goat Anti-Mouse IgG monoclonal, HRP-conjugated anti-mouse IgG polyclonal (#501240, 1:10, Cayman chemical)
Validation Refs. from the manufacturer's datasheet: https://www.caymanchem.com/product/501240

Mouse anti-IgM monoclonal, HRP-conjugated anti-mouse Ig(H+L) polyclonal (#88-50470-22, 1:250, Thermo Fisher Scientific)
Validation Refs. from the manufacturer's datasheet: https://www.thermofisher.com/elisa/product/IgM-Mouse-Uncoated-ELISA-Kit-with-Plates/88-50470-22

Eukaryotic cell lines

Policy information about cell lines and Sex and Gender in Research

Cell line source(s)	Lung organoids were prepared from human lung tissues harvested with the patients' consent.<br>HEK293T-Rspo1-Fc cells were purchased from Calvin Kuo's Laboratory at Stanford University.<br>A human induced pluripotent stem cell (hiPSC) line (CHO) was non-commercial and provided by the Yonsei University School of Medicine. Hepatic endodermal cells and hepatic stellate cells were differentiated from hiPSCs.<br>Human umbilical vein endothelial cells (HUVECs) and human mesenchymal stem cells (hMSCs) were purchased from Lonza.<br>HEK293T cells were ordered from American Type Culture Collection (ATCC).
Authentication	Lung organoids were authenticated with immunostaining of airway markers (P63, MUC5AC, α-tubulin).<br>HEK293T-Rspo1-Fc cells were not authenticated after purchase.<br>hiPSCs were authenticated with immunostaining of pluripotency markers (OCT4, TRA-1-60, SOX2) and alkaline phosphatase staining.<br>HUVECs and hMSCs were authenticated by Lonza before delivery and not authenticated subsequently.<br>HEK293T cells were authenticated by ATCC before delivery using short tandem repeat (STR) profiling.
Mycoplasma contamination	hiPSCs were regularly checked and negative for mycoplasma contamination.<br>HUVECs and hMSCs negative for mycoplasma contamination were purchased and not authenticated subsequently.<br>HEK293T-Rspo1-Fc cells were not authenticated after purchase.<br>HEK293T cells were certified not contaminated by ATCC and not tested from mycoplasma contamination subsequently.
Commonly misidentified lines<br>(See ICLAC register)	No misidentified cell lines were used in this study.

Animals and other research organisms

Policy information about studies involving animals; ARRIVE guidelines recommended for reporting animal research, and Sex and Gender in Research

Laboratory animals	Male C57 mice aged 4-8 weeks and Male Balb/C mice aged 4 weeks were used for in vivo experiments. Animal housing room temperatures are monitored at all times and maintained between 71 and 75 degrees F for most species according to their physiological needs. Humidity is maintained between 30-70%. Light intensity and light cycle timing are carefully regulated and monitored in Caltech laboratory animal facilities. Automated light timers ensure a consistent light-dark cycle with 13 hours on and 11 hours off.
Wild animals	This study did not involve wild animals.
Reporting on sex	This study did not involve sex specificity in the study design.
Field-collected samples	This study did not involve samples collected from the field.
Ethics oversight	Institutional Animal Care and Use Committee (IACUC) of the California Institute of Technology (Caltech) for animal experiments.

Note that full information on the approval of the study protocol must also be provided in the manuscript.
